# Soybean cyst nematode detection and management: a review

**DOI:** 10.1186/s13007-022-00933-8

**Published:** 2022-09-07

**Authors:** Youness Arjoune, Niroop Sugunaraj, Sai Peri, Sreejith V. Nair, Anton Skurdal, Prakash Ranganathan, Burton Johnson

**Affiliations:** 1 School of Electrical Engineering and Computer Science (SEECS), University of North Dakota, Grand Forks, USA; 2 Department of Aviation, University of North Dakota, Grand Forks, USA; 3 Plant Sciences, North Dakota State University, Fargo, USA

**Keywords:** Soybean, Soybean cyst nematode, Machine learning, Deep learning, *Heterodera glycines*, Data augmentation, Convolutional neural networks, Hyperspectral imaging, Multispectral imaging, Vegetation indices

## Abstract

Soybeans play a key role in global food security. U.S. soybean yields, which comprise $32\%$ of the total soybeans planted in the world, continue to experience unprecedented grain loss due to the soybean cyst nematode (SCN) plant pathogen. SCN remains one of the primary disruptive pests despite the existence of advanced management techniques such as crop rotation and SCN-resistant varieties. SCN detection is a key step in managing this disease; however, early detection is challenging because soybeans do not show any above ground symptoms unless they  are significantly damaged. Direct soil sampling remains the most common method for SCN detection, however, this method has several problems. For example, the threshold damage methods—adopted by most of the laboratories to make recommendations—is not reliable as it does not consider soil pH, N, P, and K values and relies solely on the egg count instead of assessment of the root infection. To overcome the challenges of manual soil sampling methods, deep learning and hyperspectral imaging are important current topics in precision agriculture for plant disease detection and have been proposed as cost-effective and efficient detection methods that can work at scale. We have reviewed more than 150 research papers focusing on soybean cyst nematodes with an emphasis on deep learning techniques for detection and management. First: we describe soybean vegetation and reproduction stages, SCN life cycles, and factors influencing this disease. Second: we highlight the impact of SCN on soybean yield loss and the challenges associated with its detection. Third: we describe direct sampling methods in which the soil samples are procured and analyzed to evaluate SCN egg counts. Fourth: we highlight the advantages and limitations of these direct methods, then review computer vision- and remote sensing-based detection methods: data collection using ground, aerial, and satellite approaches followed by a review of machine learning methods for image analysis-based soybean cyst nematode detection. We highlight the evaluation approaches and the advantages of overall detection workflow in high-performance and big data environments. Lastly, we discuss various management approaches, such as crop rotation, fertilization, SCN resistant varieties such as PI 88788, and SCN’s increasing resistance to these strategies. We review machine learning approaches for soybean crop yield forecasting as well as the influence of pesticides, herbicides, and fertilizers on SCN infestation reduction. We provide recommendations for soybean research using deep learning and hyperspectral imaging to accommodate the lack of the ground truth data and training and testing methodologies, such as data augmentation and transfer learning, to achieve a high level of detection accuracy while keeping costs as low as possible.

## Introduction

**Fig. 1:**
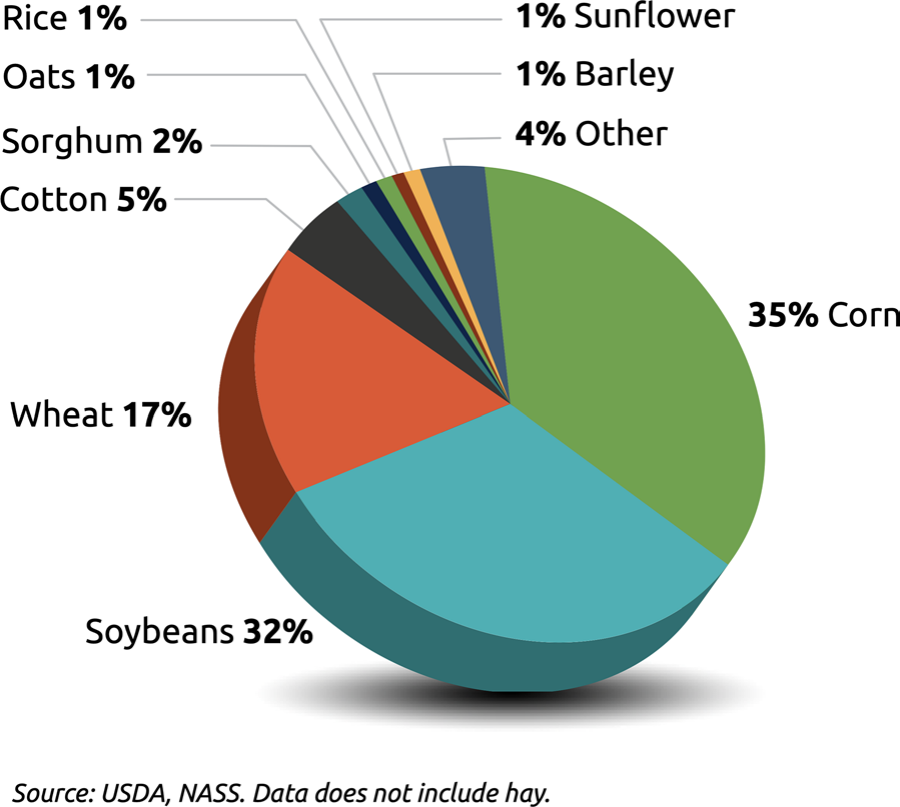
Crop areas in 2021

The soybean is one of the most important legume crops produced globally and is particularly vital in the United States of America (Fig. 1). This crop is an important agricultural commodity and source of revenue since it is one of the world’s largest animal protein feed sources of and is the second largest vegetable oil source. The U.S. is the world’s leading soybean producer and second major exporter: soybeans encompass 32% of the total planted crop area according to a 2021 USDA report. Soybeans produced in the Midwestern Corn Belt region strengthened the 2017 U.S. economy by US $33 billion with more than 98 million Mg of soybeans (USDA NASS 2018). The total soybean sector contribution to the US economy averaged $$\$115.8$$ billion per year.

**Fig. 2:**
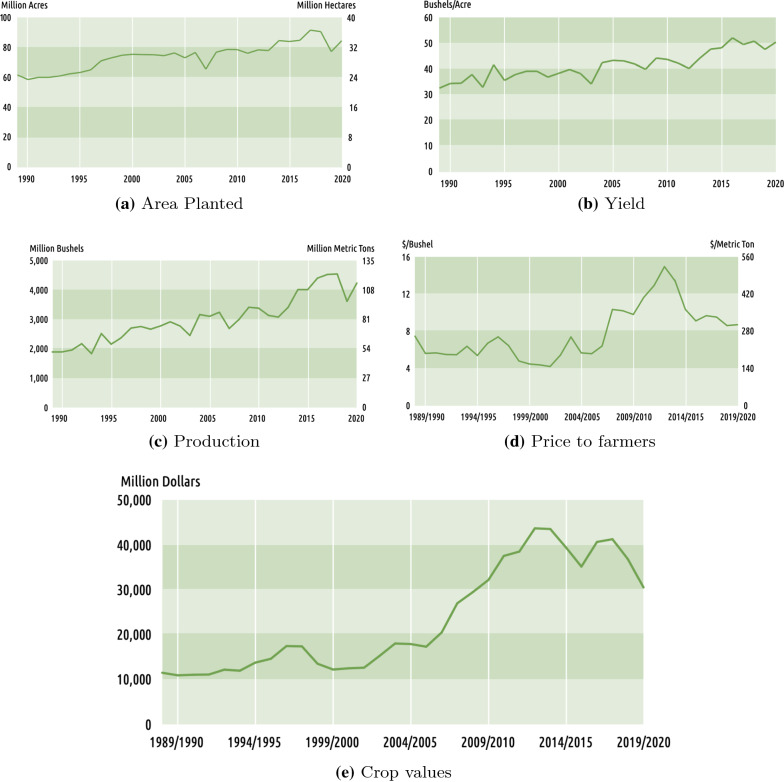
US soybean statistics 1988–2020

**Fig. 3:**
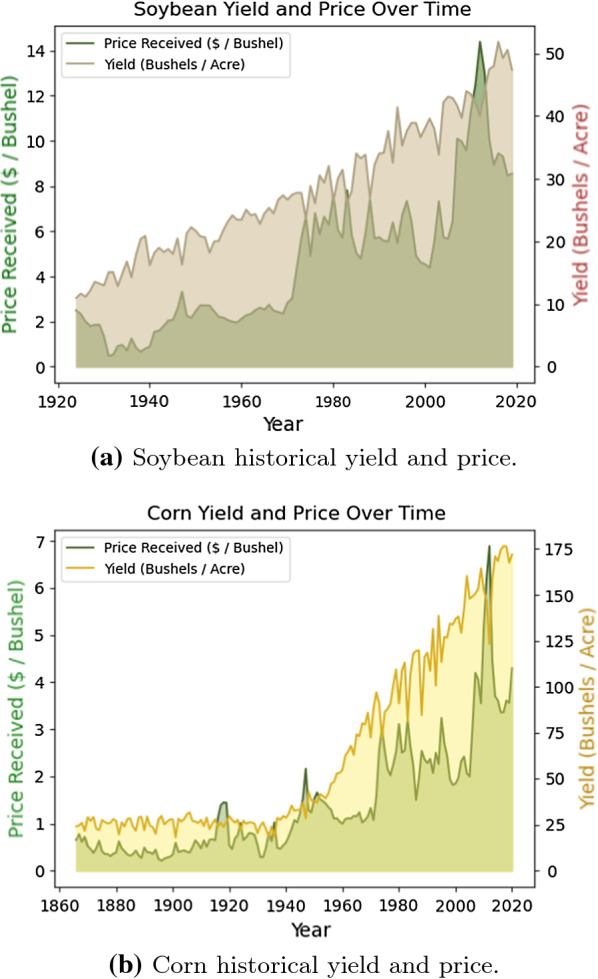
US soybean and corn prices and yields till 2020

**Fig. 4:**
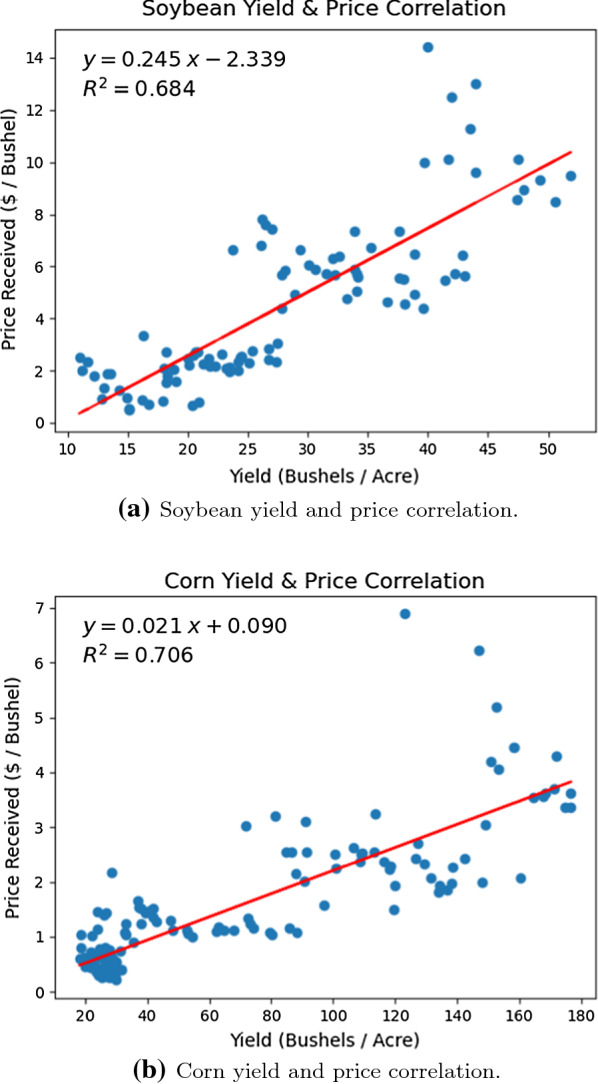
US soybean and corn yield and price linear regression analyses

**Fig. 5:**
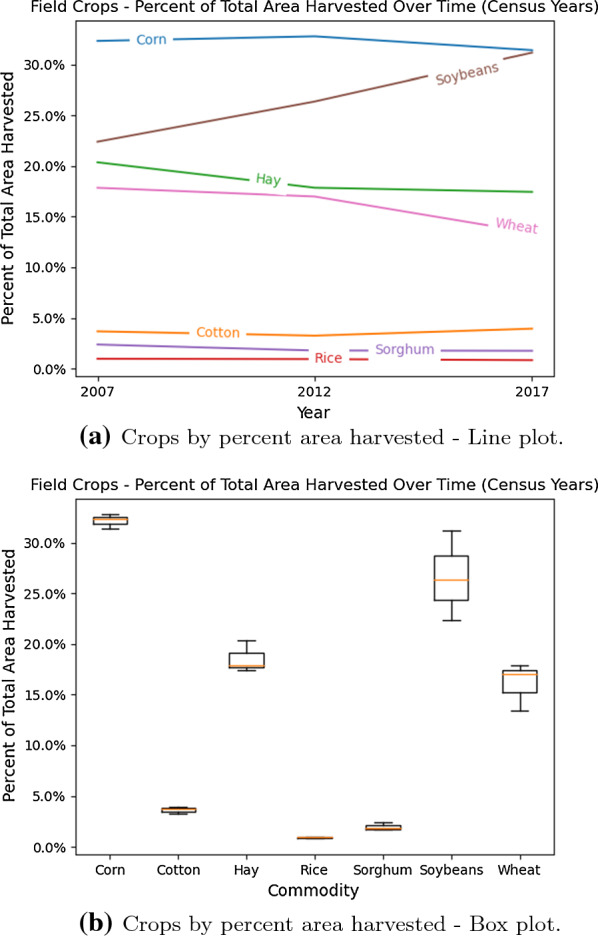
Crops harvested based on area for census years (2007–2017)

Figure 2 indicates U.S. Soybean 1989–2020 total planted area. This figure indicates that in 2020 more than 32 million hectares of the soybean crop were planted. For the same year, the yield is 50 bushels/acre (Fig. 2b) and the production is about 108 million bushels (Fig. 2c). Given that the price to farmers is about $280 per metric ton (Fig. 2d), the resulting total crop value is $30 million.

**Fig. 6:**
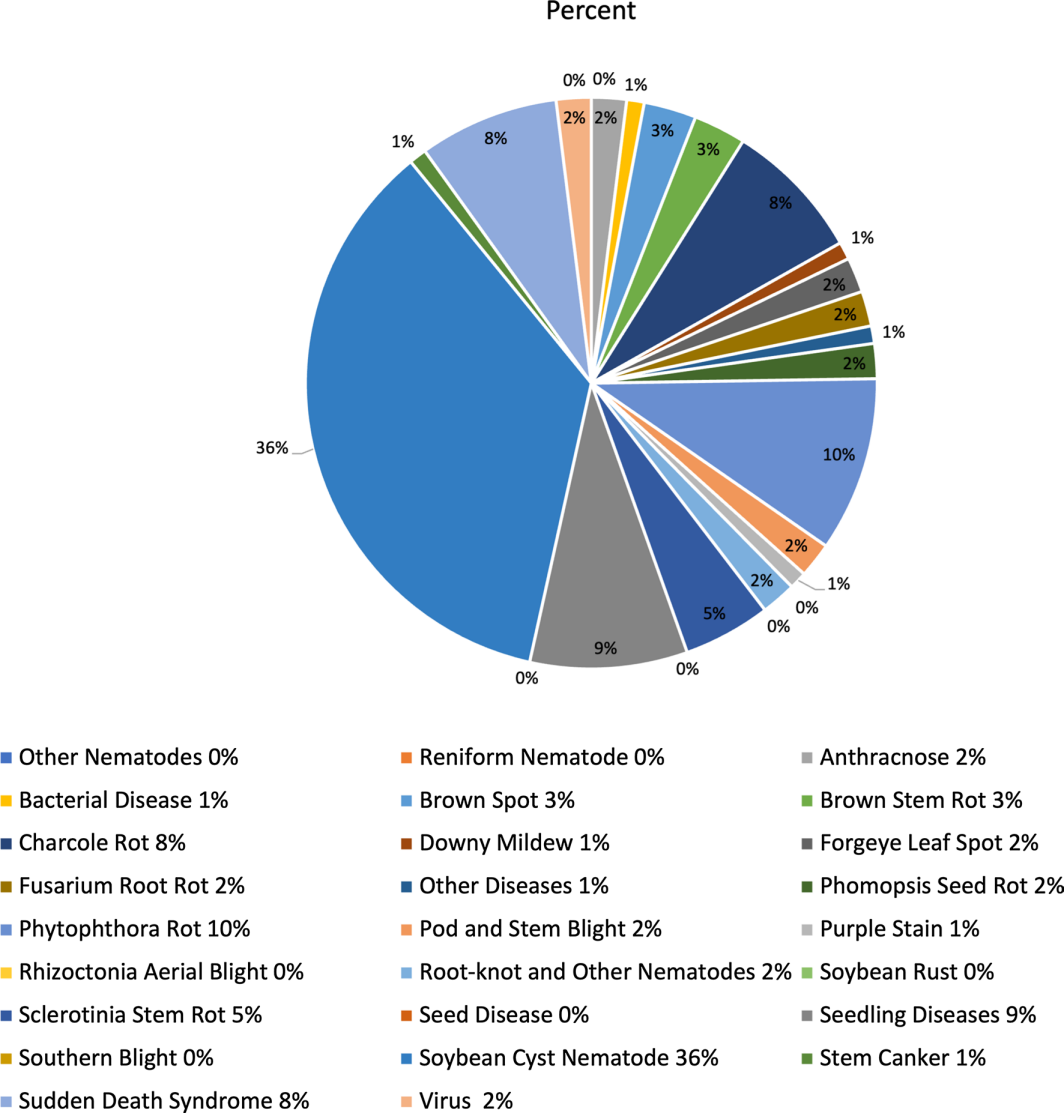
Diseases causing soybean yield reduction in USA between 1996 and 2014

*Heterodera glycines*, also known as soybean cyst nematode (SCN), is one of the most disruptive plant pathogens known to soybean crops and drastically reduces the harvested soybean yield. Once a soybean field is infected with SCN to a high degree, it is challenging to eliminate it from the field completely. The infections growth and subsequent spread can be controlled through the planting and rotation of non-host crops, planting resistant soybean seed varieties, and the proper cleaning and maintenance of the equipment and gear used within SCN infested fields. Non-host crops, such as alfalfa, oats, corn, sorghum, or wheat, are most commonly rotated with the soybean crop. Figures 3, 4, and 5 compare the soybean and corn crops with regards to historical yield and price, and contrast the harvest areas for 7 major crops for the census years between 2007 and 2017. The most common resistant soybean crop varieties planted include P188788, Hartwig, and CystX. Soybean seed variety resistance does not simply mean that the plant is immune to SCN, it implies that the plant has enhanced capabilities that reduce SCN formation at its roots. If the same SCN resistant variety is used for several years, it becomes less effective since SCN mutates to enable it to infect the resistant variety. Figure 6 shows the yield loss caused to soybean crops due to various pathogens or plant defects between 1996 and 2014. Losses due to SCN holds the largest share at 36%.

**Fig. 7:**
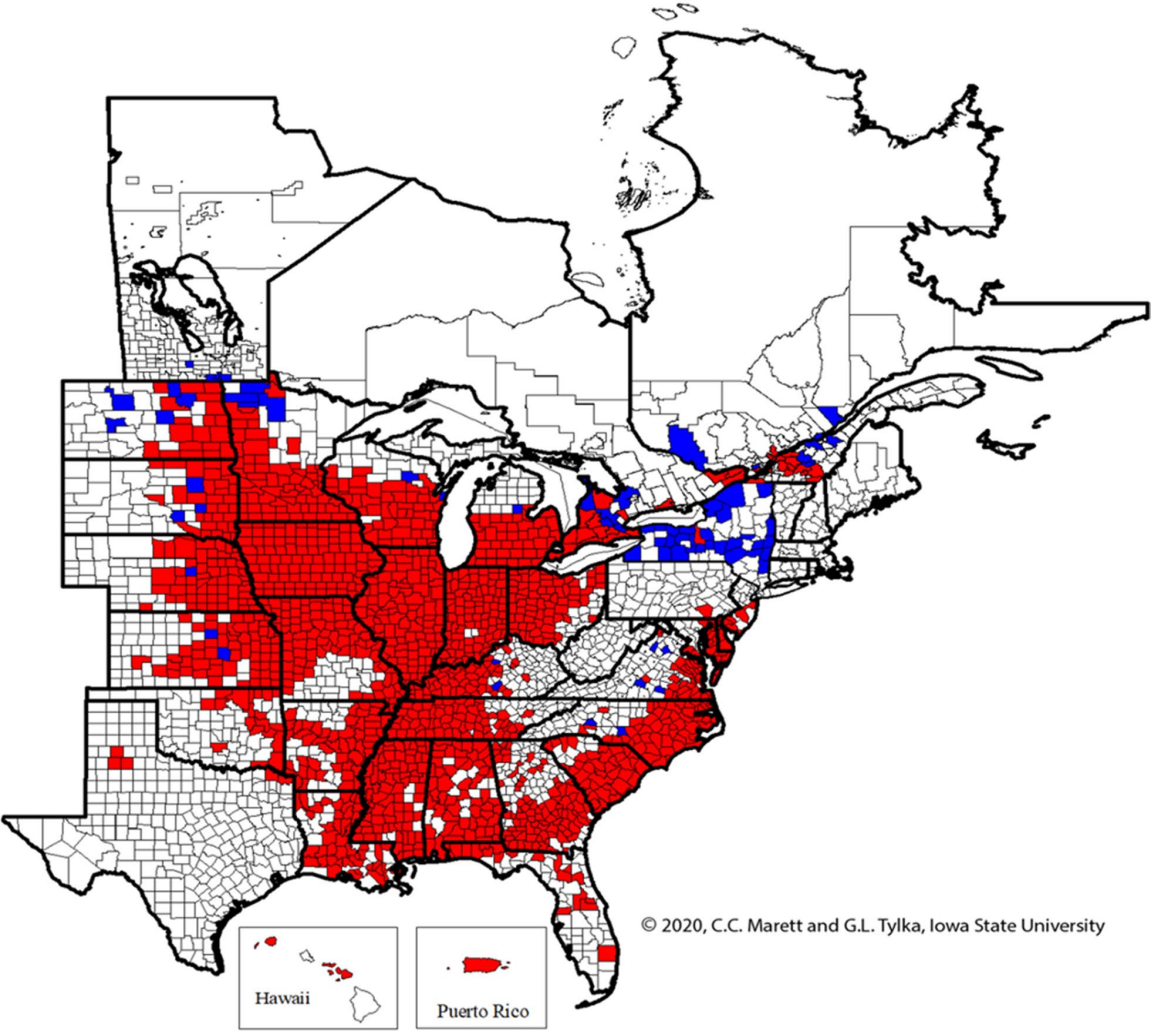
Map showing the known distribution of SCN in US-East Central, US-Southeast, US-Midwest, parts of US-Heartland and US-Southwest and Canada [1]

**Fig. 8:**
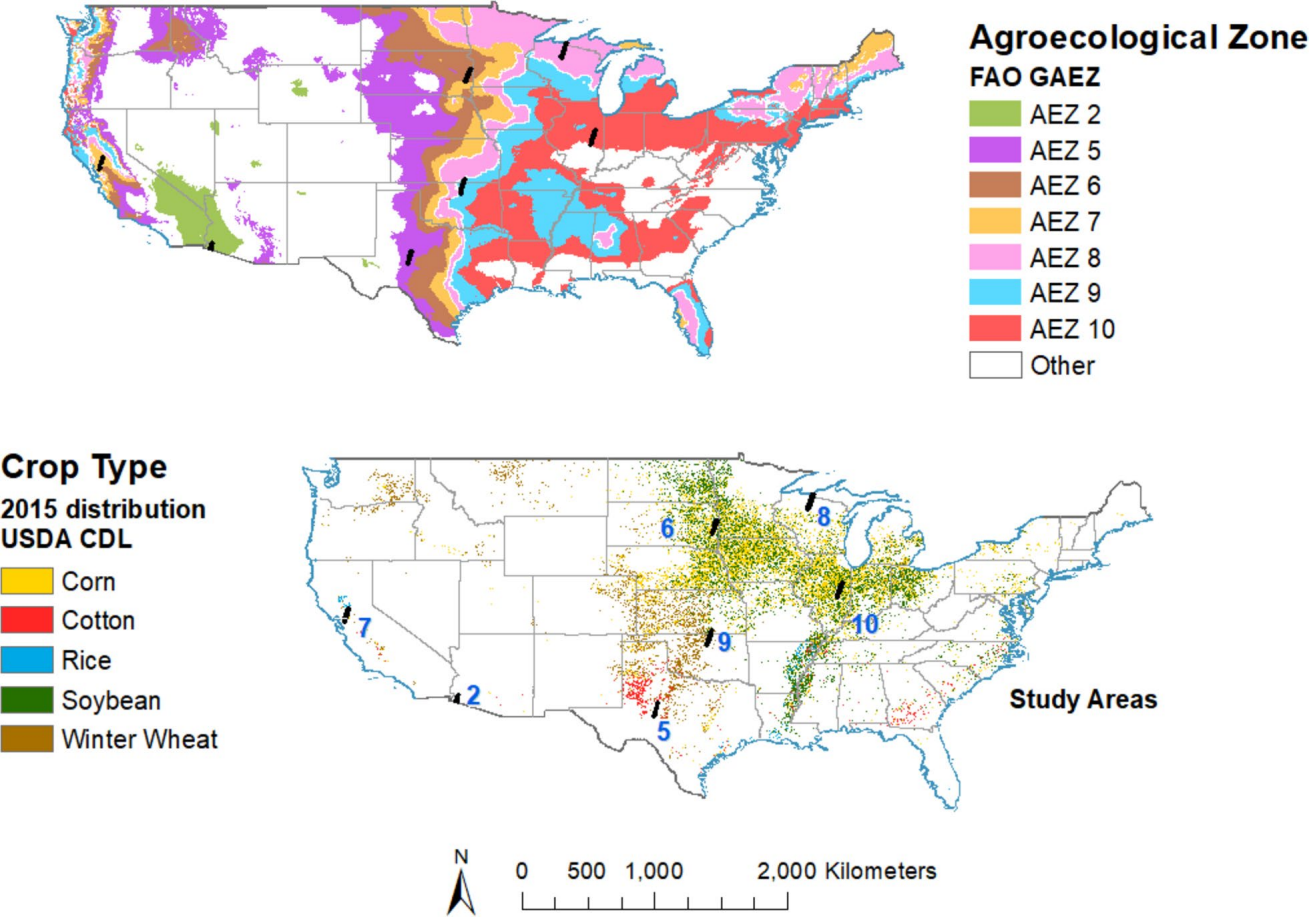
Global agro-ecological zones and crop type distribution in USA [2]

SCN was first found in North Carolina in 1954. The disease was subsequently identified throughout the northwestern and southeastern states of the US. Figure 7 presents the 2020 North American SCN distribution. Figures 7 and 8 together illustrate that most of the states in the US are infested with SCN. SCN spreads by anything that moves soil, including wind, water, machines, and living creatures, especially birds. SCN can multiply rapidly in the presence of a host plant even though movement occurs slowly. SCN can live for many years in the soil without the support of a host plant. It is relatively difficult to recognize an SCN infested field visually, especially when damage is low. Understanding SCN biology and plant behavior is key to recognizing early infection and managing negative economic impact. SCN is the main sources of yield loss: it accounts for up to 30% of all US soybean yield lost to disease, amounting to a little over 2.7 million metric tons a year. The most recent estimates indicate that SCN infestations resulted in $1.5 billion in annual yield losses . The estimated reduction of US soybean yields due to SCN in 2005 was 1,935,493 tons [3]. The average economic loss due to soybean disease was estimated at $60.66 USD per acre between 2010 and 2014 in the Unites States and Ontario [4].

Figure 7 also indicates that SCN was detected for the first time in more than 50 counties in the US and Canada (Fig. 7, red) before spreading to more states and counties (Fig. 7, blue). Figure 8 depicts crop type distribution in the USA, indicating that most soybean farms are located in mid-eastern regions.

**Fig. 9:**
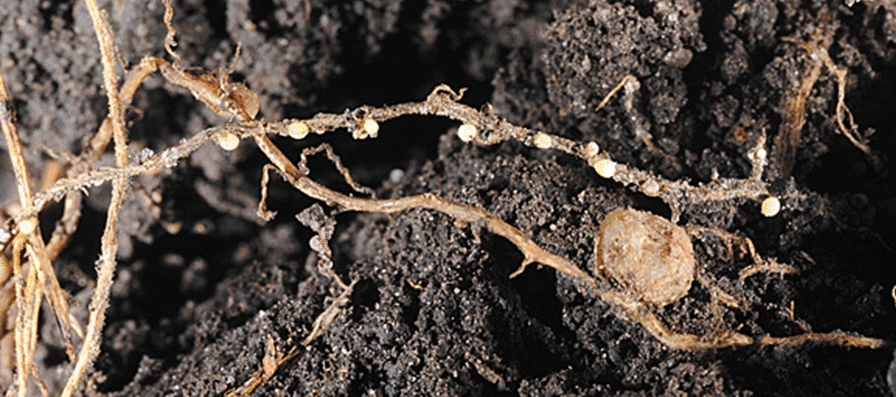
Cream-colored cysts and one nodule on soybean roots. (Sam Markell, NDSU)

**Fig. 10:**
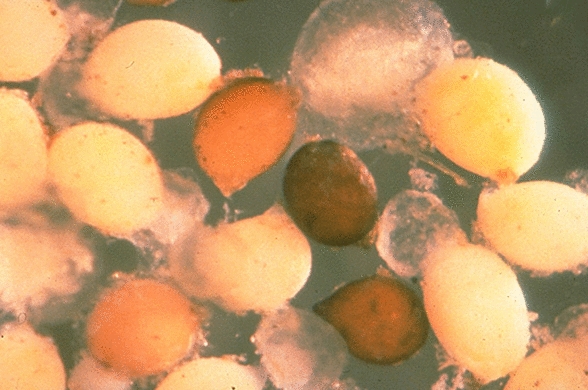
Cream-colored live female cysts and brown cysts of dead females. (Photo courtesy of Terri Niblack, University of Illinois)

**Fig. 11:**
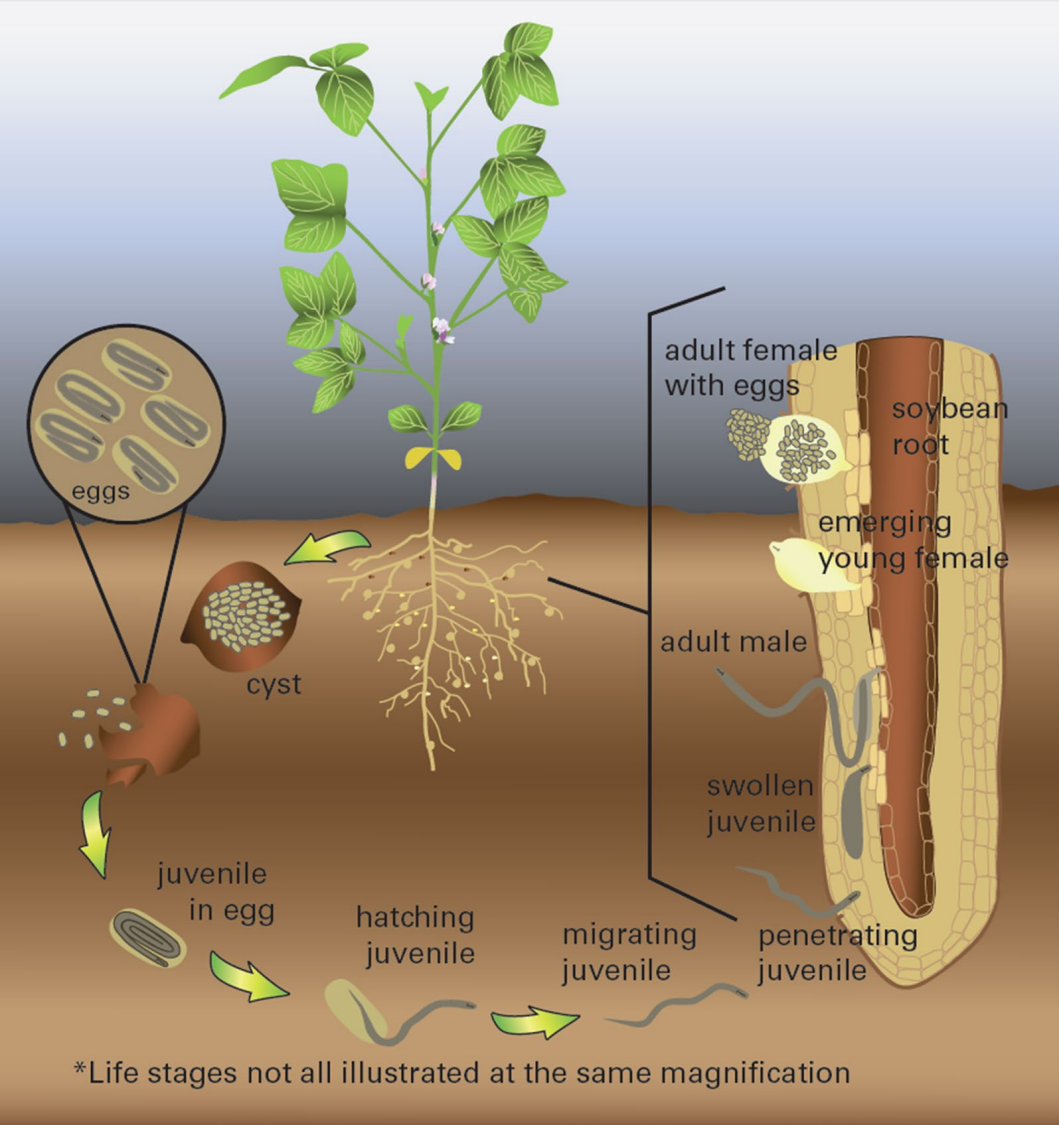
SCN life cycle [6]

Infection begins when a juvenile nemotode enters the soybean root and feeds on the cells in the root’s vascular system. A male juvenile will move into the soil once it has finished feeding; therefore, it does not cause as much infection as a female since females do not migrate but rather, mature in place. The young adult female is white in color and is often visible to the naked eye in the soybean field when the plant begins to flower. As the adult female ages, its color changes to yellow and then brown. The brown stage of the cyst can contain up to 500 eggs and can persist for years in a dormant state. The white and yellow female stages are the only visible sign of SCN infection on the roots and may not be present at the time of sampling [5] (please see Figs. 9 and 10)as the brown cysts are too small and are not visible in oil. The typical SCN life cycle begins in the spring, which is the planting season for the soybean crop (Fig. 11). Temperatures and moisture levels during this season are suitable conditions for the hatching of the eggs released by the cysts, or the dead female nematodes. It takes 24–30 days for the eggs to hatch and release juvenile nematodes. These juvenile nematodes infect the soybean plant’s vascular tissue. Female nematodes can be observed at the plant’s roots approximately 6 weeks after soybean seeds are planted. Some of the symptoms of this disease include severe growth retardation, stunting, and a yellowish appearance. Specific questions relevant to soil sampling, such as grid spacing and sampling frequency, cannot be definitively addressed since there many factors that need to be considered, such as topography, soil type, and fertilizer use; however, a general framework can be used as an initial step.

Soybean vegetative and growth stages are depicted in Table 1. There are five vegetation stages and eight reproduction stages. The vegetation stage last 43 days on average while reproduction stage last 73 days. There are 7 sub-stages in the vegetation stage: VE, VC, V1, and through V5, while there are eight sub-stages for the reproduction stage: R1 through R8. Researchers have defined the beginning of the vegetative stages as when the soybean plant emerges from the soil. These stages are identified based on how many nodes are unifoliolated. The unifoliolate nodes are counted as one node even though there are two separate nodes that occur at the same position and time. V1 corresponds to one trifoliolate, V2 corresponds to 2 triofoliolates, and so on. The reproduction stages are identified by the beginning of flowering stages where R1 corresponds to flowering onset and R8 corresponds to full maturity. The diseased plant’s foliage also falls off early and only bears a few flowers and seeds, which results in reduced size and quality. The affected plant’s root carries several lateral rootlets, which bear fewer bacterial nodules in contrast to those of uninfected plants. The disease first appears in circular patches on the field before it spreads continuously throughout the season. It takes approximately two to three years to cover the whole field if the soybeans are planted on infested land. These symptoms are often caused by other reasons; however, with some expertise, it is not hard to recognize SCN’s presence occurrence in the field.

**Table 1:** Soybean vegetation stages and duration [7]

Vegetation stage	Description	Range and average
	Planning Emergence: the cotyledon (VE) stage	5–10 days (Average 10 days)
	VC: Unrolled unifoliolate leaves	3–10 days (5 days on average)
	V1: 1st trifoliolate	3–10 days (5 days on average)
	V2: 2nd trifoliolate	3–10 days (5 days on average)
	VN Nth trifoliolate	3–10 days (5 days on average) *V*3 3–8 days (5 days on average) for *V*5 3–5 days (3 days on average) for beyond *V*5
	R1: Beginning flower	2–5 days (3 days)
	R2: Full flowering	0–7 days (7 days)
	R3: Beginning pod	5–15 days
	R4: Full pod	4–26 days
	R5: Beginning seed	11–20 days
	R6: Full seed	9 to 30 days
	R7: Beginning maturity	7–18days
	R8:Full maturity	–

Some clear above-ground SCN infestation symptoms are lower yields, stunting, and crop cover yellowing [8] either circular or oval shaped. Another minor but important detail that can be overlooked is that SCN infested crop height is lower than healthier plants [9]; however, the most effective way to diagnose SCN infection is through soil tests [10]. It is nearly impossible to detect SCN affected plants or soils with the naked eye. The above ground symptoms are often misleading and the level of infection is difficult to estimate by simply observing the white female attached to the soybean root.

The current method used to determine the level of infection is through soil testing done via professional diagnostic laboratories. Soil can be sampled at any time to check for the presence of SCN in a field. Ideally, soil samples should be analyzed in the fall before the soybean harvest to detect the level of SCN infection since this is when most of the SCN females mature. Atleast one sample for each 10-acre field should be analyzed [11]. Typically, multiple steps of sieving, rinsing, grinding, and cleaning the soil samples are repeated to extract SCN cysts and count the number of eggs. This is laborious and requires the expertise of trained professionals to carefully handle the samples for cyst extraction. However, a recent work by Legner and colleagues [12] automate this process of extraction and soil analysis by using a “robotic agricultural instrument” equipped with elutriators and robotic handlers. This instrument takes 4 min to process 100 cc of soil as compared to the typical extraction style which takes about 10 min for extraction. Plants located in the center of the SCN affected region have several stunted root systems that are severely distorted, swollen, and have lumps known as root knots. The below ground symptoms include increased susceptibility to other soil borne plant pathogens. The above ground symptoms include stunted plants, mid-season yellowing, and premature senescence, or aging; however, SCN infection symptoms are not always visible above-ground. Yellowing can be caused by other diseases, but the timing of the yellowing caused by the cyst nematodes often start to appear one month after planting in July and August. There are several factors that contribute to SCN development and its subsequent growth, including soil dispersing agents, the presence of host crops, adaptability to SCN-resistance, management practices, and soil properties.

**Fig. 12:**
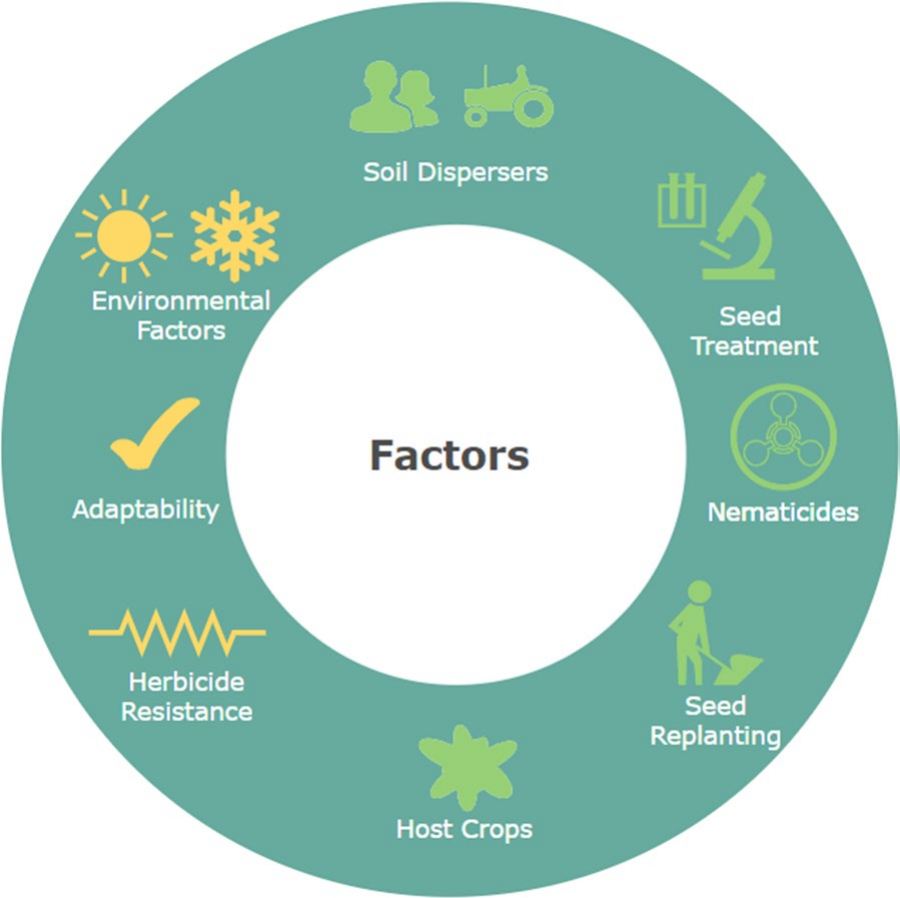
Factors directly affecting SCN development

Figure 12 depicts the eight factors affecting SCN development: host crop usage, soil dispersers, seed treatments, nematicides, seed replanting from infected soils, environmental factors (temperature, moisture, pH), SCN herbicide resistance, and adaptability to SCN-resistant varieties. The factors marked in yellow are ambient factors such as temperature and humidity, SCN adaptability to the planted seed variety, and SCN resistance to applied herbicides. These factors are beyond human control. The factors marked in green, such as soil dispersing agents like farm machinery, seed treatment measures taken to prevent SCN adaptability, nematicide usage to slow SCN growth, seed replanting from infested fields, and host crop rotation with crops such as oat, alfalfa, and wheat are within human control. Any visible SCN development signs can only be identified in vegetation stage V4 when the fourth trifoliolate occurs [13]. The soils used to grow soybeans have a bearing on SCN growth [14]. This factor was not included in Fig. 12 since further research needs to be conducted to verify the effects of different soil types on SCN growth or decline. Commonly used soil types and their respective characteristics are:

1. Sand: Larger particles size, low nutrients, acidic, and easy moisture drainage.
2. Silt: Small particle size, more nutrient dense than sand, less acidic than sand, more moisture content than sand.
3. Clay: Smaller particle sizes, nutrient dense, alkaline, and poor moisture drainage.
4. Loam: A combination of sand, silt, and clay textures. Nutrient rich, drought resistant, alkaline, and ideal for agriculture.

These soil types, along with ambient factors such as temperature, pH, and humidity, promote or inhibit SCN growth. Irrigated soils, or soils with higher moisture content, have larger SCN populations than non-irrigated soils [15]. Experimental work that highlights the influence of abiotic factors such as temperature, pH, soil type, and soil moisture and their influence on soybean egg count (EC) is not common in the literature, but two  key findings have been identified: at temperatures above 98.6 $^{\circ }$F in a wet or dry clay contributed to a lower EC [14, 16], while an increased EC was identified in a wet loam environment at a lower temperature of 77 $^{\circ }$F [14, 15, 17].

Researchers have been analyzing different methodologies to improve soybean yield and reduce the loss associated with soybean diseases such as SCN. Different SCN detection methods and management mechanisms, as well as the use of SCN resistant varieties have been developed. Most SCN detection techniques can be sorted into two groups: soil sampling methods and computer vision methods. Soil sampling methods consist of taking soil samples from areas where the soybean plants are experiencing yield loss and counting egg density. Computer vision uses remote sensing to detect infested fields. Remote sensing consists of using imaging combined with earth surface reflectance spectra to detect anomalies. These images can be acquired acquired using satellites, aircraft, or drones. For more information, the authors of [18] proposed a review of hyperspectral image classification using deep learning. These collection methods can work at scale but an equally important step is to consider SCN management methods to control (not eliminate) SCN growth.

**Fig. 13:**
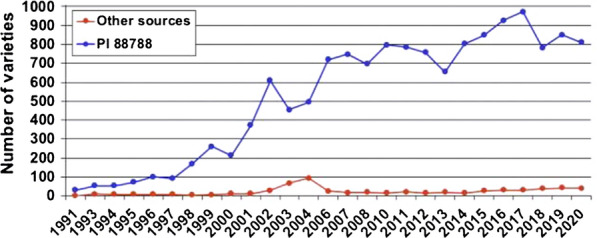
The use of PI 88788 vs other types [20]

Several management techniques are considered when taking action against SCN. A recent survey indicated that 49% of soybean farmers now rotate genetic SCN resistance sources, of which 25% are using Peking as an alternative SCN resistance source. An identical survey conducted in 2015 indicated that 39% of soybean farmers were rotating genetic SCN resistance sources, and of those farmers, 95% are using the SCN resistant PI 88788. Over 95% of these plants are sourced from PI 88788 [19]. Figure 13 indicates that PI 88788 was the most used SCN resistant crop from 1991 to 2016. SCN resistant varieties overuse have created a new problem where several soybean field showed that SCN developed resistance against SCN resistant sources. Midwestern states and the respective percentages of virulent phenotypes in are Missouri at 78%, Kansas at 64%, Indiana at 56%, and Wisconsin at 78%. Rotating SCN with some other non-host crop is a practice applied by soybean farmers to mitigate SCN. At least 77% of soybean farmers were rotating non-host crops such as corn and wheat in 2020, up from 71% in 2015. More than 60% of soybean farmers were planting SCN-resistant soybean varieties; therefore, the authors of [19] investigated novel resistance sources to soybean cyst nematodes (SCN) in wild soybeans.

**Fig. 14:**
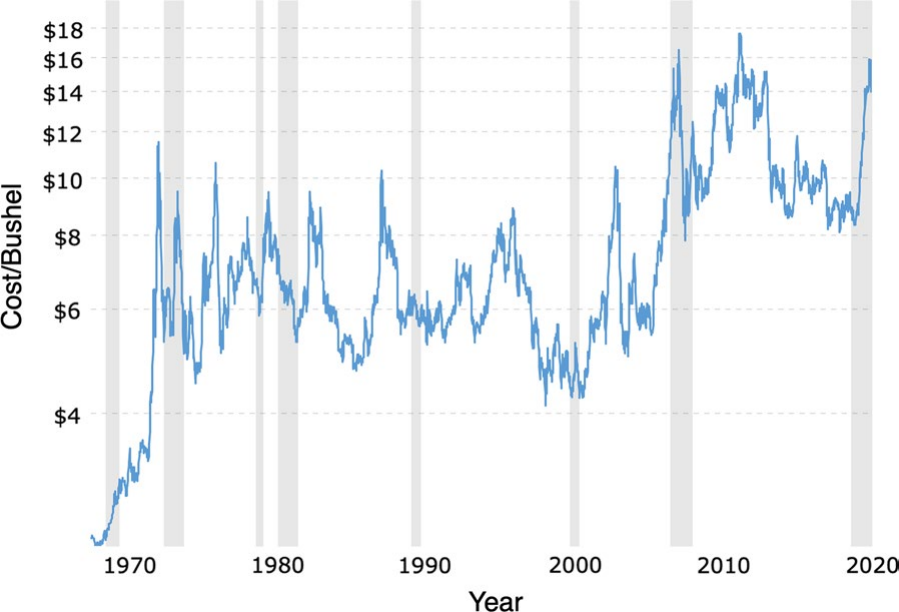
Soybean prices—Historical macrotrends (2021-06-19) [21]

The price of soybean in 2020 according to the chart shown in Fig. 14 is about $\$16$ per bushel, a 5.1-bushel-per-acre yield loss represents leaving $\$81.6$ per acre in the field. In addition, if the price remain high, farmers tend to grow soybean and this can make SCN management more difficult.

### Related work

The state-of-the-art recent review papers review remote sensing, machine learning (ML), and deep learning; however, other papers reviewed SCN for plant diseases or precision agriculture are shown in Table 2. Few to no review papers exist that provide in-depth studies of SCN detection and management strategies with a focus on computer vision. The authors of [1] reviewed the states and the counties where the SCN is distributed. The authors of [22] provide a survey of SCN population densities and virulence phenotypes during 2015–2016 in Missouri. The authors of [23] provide a survey of SCN distribution in North Carolina in 2017. The authors of [24, 25] reviewed the role of remote sensing in precision agriculture. The authors of [26] surveyed the use of unmanned aerial vehicle (UAV) sensing in precision agriculture. A brief survey of hyperspectral sensing application in remote sensing is provided in [27]. The authors of [28] proposed a survey of public datasets that can be used for precision agriculture. The authors of [29] proposed a survey of unsupervised ML  techniques for precision agriculture. The authors of [30] proposed a survey of supervised ML  classifiers for plant disease detection. The authors of [31] provided a survey on ongoing research related to computer vision, IoT, and data fusion for crop disease detection using ML  techniques. The authors of [32] proposed a review of image-based plant disease detection, focusing on ML  and deep learning. The authors of [33] provided a survey on the recent findings on the genes that control SCN resistance in soybeans. There are few papers that focus on recent advances in soybean cyst nematodes detection using remote sensing, computer vision, and ML . We will provide a comprehensive survey of SCN detection techniques that focus on direct and indirect methods, such as ML  and computer vision combined with imaging, to detect SCN, classify different seeds, determine irrigation levels, and forecast soybean yield loss.

**Table 2:** Recent review papers

References	Paper type	Topics covered	Field	Year
[31]	Review	CV, IoT, data fusion, ML	Crop disease	2021
[30]	Review	ML	Plant disease detection	2019
[29]	Review	Unsupervised ML	Precision agriculture	2015
[28]	Review	Public dataset	Precision agriculture	2020
[26]	Review	UAVs sensing	Agriculture precision	2019
[24]	Review	Remote sensing	Precision agriculture	2010
[25]	Review	Remote sensing	Precision agriculture	2017
[1]	Review	SCN distribution	Soybean disease	2021
[22]	Review	Population densities and virulence phenotypes	Soybean disease	2018
[32]	Review	ML, DL, Imaging	Plant diseases	2021
[33]	Review	SCN resistant varieties	SCN	2018
[34]	Review	Breeding, genetics, and genomics	Soybean nematode species	2016

### Motivation and contributions

SCN detection and management is challenging with some of these challenges being:

- Lack of early SCN detection because above ground foliage damage does not appear until infection levels reach highly damaging levels. The infection becomes very difficult to manage because of the steady increase in egg density.
- Once a soybean field is infested with SCN, it takes several years to disappear completely as SCN eggs stay intact for about several years as the cyst protects them even with consecutive non-host crop rotations [35].
- It is challenging to draw conclusions regarding the correlation between SCN egg densities and soybean yield loss.
- SCN persists and spreads in fields with diverse environments.
- Reproduction capacity of SCN is high [36].
- Corn and soybean rotations are ubiquitous in the US.
- High level of diversity among virulent phenotypes.
- A shift in soybean cyst nematode virulence is associated with the use of soybean resistant PI 88788 [37]. A survey conducted in 2005 indicated that 83% of the soybean hectarage in Illinois is infested with SCN, with average population densities high enough to cause significant yield suppression (2700 eggs/100 cm$^{3}$ soil) [37], while 70% have SCN in these fields that have adapted to PI 88788 to some extent.
- One observation may not be enough: SCN does not necessarily cause symptoms that can be detected visually unless the damage has reached high levels [17].

With these challenges comes the need for robust solutions that leverage artificial intelligence for efficient SCN detection. Deep learning-based hyperspectral image analysis is gaining popularity because of the advantages this methodology presents over direct soil methods. The use of direct soil methods requires large amount of time to count the eggs and sampling fields, and may not yield information about the existence of soybean cyst nematodes at an early stage. The lab tests indicate that the number of eggs sampled from the field is only an approximate. It is often challenging to determine the best area to obtain samples from since SCN densities will vary between areas of an infested field. Some recommendations suggest sampling from places where visible symptoms are present; however, in most of the cases the symptoms are not visible at early stages. Deep learning provides an alternative to these testing methods: hyperspectral images from the whole field are obtained and then the  AI model can use segmentation techniques to indicate the presence of cyst nematodes and which parts of the field are infested. Deep learning, combined with UAVs equipped with hyperspectral imaging capabilities, can present an opportunity to obtain hyperspectral images on a regular basis and provide more details about soybean plant health.

## Current soybean cyst nematode detection approaches

**Fig. 15:**
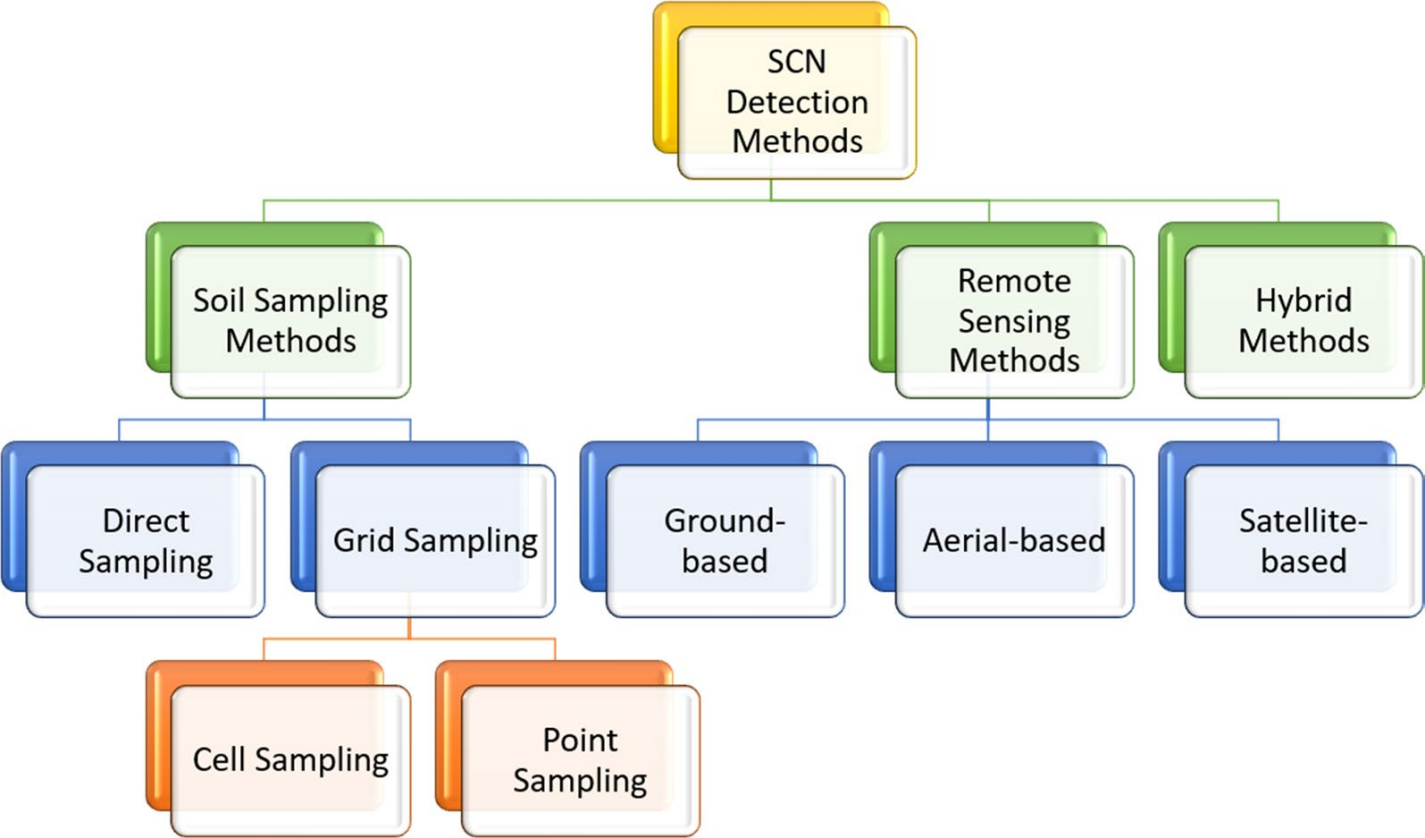
Simple taxonomy of SCN detection and management

Soybean cyst nematode detection techniques may be mainly grouped into: soil sampling, remote sensing, and hybrid methods (see Fig. 15). Direct soil sampling methods are based on collecting soil samples from the fields and taking that to specific labs for further analyses. This category includes sub-categories such as cell sampling, grid sampling, and point sampling. The second category is called remote sensing and consists of ground-based, aerial-based, and satellite-based methods for data acquisition. This category uses imaging techniques or wireless sensor networks (WSNs) deployed on the ground without taking soil samples. Lastly, the hybrid category combines techniques from direct and indirect detection methods.

### Soil sampling methods

Soil tests were performed in the past to primarily classify nutrient availability within a sample. Typical soil nutrients obtained from a soil test are nitrogen, phosphorus, potassium, calcium, magnesium, and sodium [38]. Out of these, nitrogen, potassium (both of which contribute to increased soybean crop yield [39, 40]), and phosphorus are the significant macro-nutrients for soybeans. Soil tests can also indicate if a soybean crop is suffering from iron deficiency chlorosis (IDC), which causes leaf-yellowing and plant stunting. Vegetable crops differ in their ability to absorb micro-nutrients such as iron from the soil. Legumes, such as soybeans, are more likely to be iron deficient [41]. There are sixteen elements that are essential in supporting optimal growth for soybean crops, according to the Mississippi Soybean Promotion Board [42]. Seven of these sixteen elements are are boron, chlorine, copper, iron, manganese, molybdenum, and zinc. These elements usually meet sufficiency levels [43] in most soils, but soil alkalinity or acidity is an equally important factor as soil pH determines nutrient absorption. As an example, phosphorus is most plant-available when soil pH is between 6 and 7 [44].

Soil sampling can be done through two ways: grid sampling or directed sampling [45, 46]. Grid sampling is the process of soil sampling every few acres, typically where multiple small fields with different crop histories have been combined into a single grid. Grid sampling creates a general but potentially less accurate nutrient map for a given area since fewer samples are collected. The alternative method, direct sampling, collects multiple samples from a specific field if the grid has different soil series, crops, or eroded areas [47]. This sampling method creates a variability map that can be verified using precision agriculture technologies such as hyperspectral or multispectral imaging. Aerial images for a given field can be used to delineate areas that are different from one another and can demarcate different subareas for direct sampling. Soil sampling involves three systematic steps:

1. Most organic matter, such as phosphorus and potassium, can be quantified using samples taken at depths as low as 0–6 inches, depending on the nutrient tests needed. Soil tests for nitrogen require samples taken at depths of 6–24 inches [47]. 6–8 inches of sampling would be ideal for most tests. Soil can be extracted using a shovel or a soil recovery probe.
2. Approximately 15–20 samples should be obtained during the spring, planting season, or in Fall, harvest, using a zig-zag or “M” pattern once the field is divided into sections [8]. Sampling soil in the fall is the most common method. Soil sampling should be avoided in wet or frozen soils.
3. Soil samples collected during step two should be combined in a bucket to obtain a composite. Bags specifically designed for soil samples should be used since sample moisture can cause cavities in regular bags.

Soil sampling was actively performed during the summer planting season (May) of 2021 jointly by the University of North Dakota (UND) and North Dakota State University (NDSU) to further identify key insights for SCN research. Two different fields, in terms of planting time and SCN population, were identified in the state of North Dakota for this research study. The first field, called Prosper, had an area of 6572 sq. m, planted with soybean seeds during the first week of May. This field had lower SCN populations based on previous field usage and crop yields. The second field, called Casselton, had an area of 3500 sq. m planted with soybean seeds approximately a week after Prosper. This field had higher SCN counts. Soil sampling was completed at certain stages of crop growth to identify SCN egg counts, pH, and micro-nutrient profiles (Tables 3 and 4). The nitrogen/phosphorus/potassium (N/P/K) levels were measured in lbs per acre and parts per million (ppm). Though the typical soil requirement (in terms of cc) is 250 cc i.e. about 1 cup, analyses for this study was done using 30 cc of soil.

It can be observed that there is a significant increase in egg counts for both the fields when comparing the measurements for July 19th and June 12th. The micro-nutrient profiles for nitrogen, phosphorus, and potassium have also seen a decrease, more substantially in the case for nitrogen and potassium. This is consistent with findings from other studies [48, 49]- which state that one of the above ground symptoms of SCN is nutrient deficiency. However, further testing may be required to conclude whether this lowered concentration of micro-nutrients is due to impaired root growth or due to the direct involvement of SCN [50] in changing the physiological characteristics of the crop.

**Table 3:** Micro-nutrient, egg count, and pH analysis for a field in Prosper, North Dakota

N/P/K	Egg count	pH	Date	Soil type
266.5/37.5/342.5	465	7.65	June 12th 2021	Heavy clay
51.4/21/185	1471	8.21	July 19th 2021

**Table 4:** Micro-nutrient, egg count, and pH analysis for a field in Casselton, North Dakota

N/P/K	Egg count	pH	Date	Soil type
260.3/42.8/400	562	7.68	June 12th 2021	Heavy Clay
87.1/40.3/273.3	5558	7.92	July 19th 2021	

### Remote sensing methods

#### Remote sensing

**Fig. 16:**
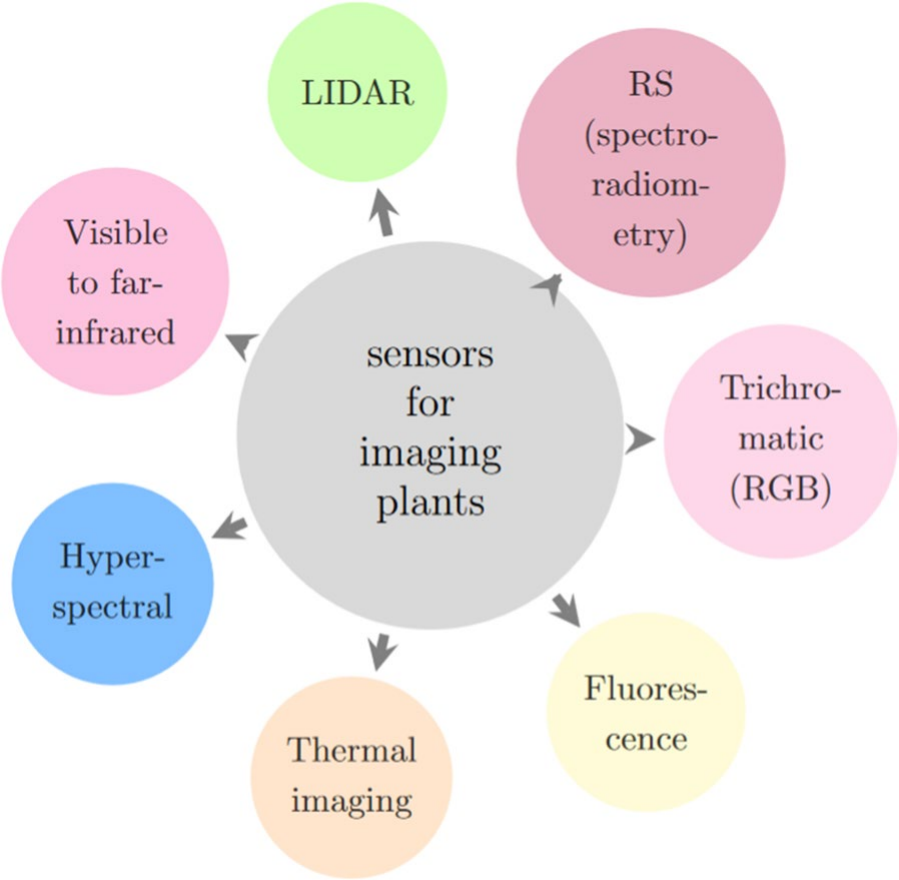
Different sensors for imaging plants

Remote sensing is the science of recording images of the earth’s surface using satellite, aircraft, and drones for analysis and interpretation. Remote sensing consists of four processes: (1) energy detection from reflected sunlight, (2) energy conversion into an electrical signal, (3) signal transmission into the ground then saved to memory, and (4) correcting and error compensation for distortions that occur during the process. Image analysis and interpretation must be completed to understand the characteristics the earth’s surface. Figure 16 shows different imaging sensors that can be used for precision agriculture.

#### Wavelengths used in remote sensing

The process of acquiring reflected sunlight energy is often performed using sensors. The required wavebands that are used for this process must be defined; therefore, atmospheric absorptions between the sunlight and the earth’s surface, and between the earth surface and the sensor mounted on the space or aircraft, must be understood. The electromagnetic spectrum primarily consists of the visible spectrum, ultraviolet, infrared (IR), radio spectrum, mmWave, and terahertz bands. The visible and near-infrared spectrum reveals some transmittance windows where the transmission is high; therefore, these are the spectra used for remote sensing. Other portions of the spectrum, such as where the waveband is higher, may also be used. Water vapor, oxygen, and other absorption features are essential to avoid low transmittance rates, all of which must be considered when designing the sensor.

#### Satellite-based remote sensing

The process of image acquisition is often completed using different platforms, such as when satellites are used to image earth’s surface. Satellites create global images, but it is hard to achieve high spatial resolution. Sensor configurations cannot be changed once the satellite is launched. Satellites travel through the complete atmospheric column, which introduces radiometric errors that must fixed once images are obtained; however, if the satellite is not moving through the atmospheric zone, these images are more stable than those obtained with aircrafts and drones.

**Table 5:** Landsat 7 spectral bands

Band#	Visible	Range	Altitude
Band 1	Visible	(0.45–0.52 $\mu$m)	30 m
Band 2	Visible	(0.52–0.60 $\mu$m)	30 m
Band 3	Visible	(0.63–0.69 $\mu$m)	30 m
Band 4	Near-infrared	(0.77–0.90 $\mu$m)	30 m
Band 5	Short-wave infrared	(1.55–1.75 $\mu$m)	30 m
Band 6	Thermal	(10.40–12.50 $\mu$m)	60 m Low gain/High gain
Band 7	Mid-infrared	(2.08–2.35 $\mu$m)	30 m
Band 8	Panchromatic (PAN)	(0.52–0.90 $\mu$m)	15 m

Satellite images such as Landsat 7 were considered for SCN detection. Landsat 7 provides eight spectral bands, including a optical and thermal band (Table 5).

Earth surface images are subject to several geometric distortions. Several sources cause geometric distortion in satellite remote sensing: the rotation of the earth during imaging, curvature of the earth when seen from space, panoramic distortion, instrumentation effect, and variations in platform altitude, attitude, and velocity. These instrumentation effects can result in under-sampling, the presence of gaps, oversampling, and overlap between instantaneous fields of view. These distortions result in image compression or expansion. Panoramic distortion is exaggerated due to earth’s curvature. These distortions must be corrected. Two approaches are used for this correction: model-based and mapping based. The first approach mathematically models distortion effects to reverse them. This approach requires knowledge of the platform position, velocity, altitude, and time. This approach can be complex since several sources can contribute to the distortions. The second approach uses mapping to avoid modeling distortions, via an available map assumed to be correct and to which the distorted image is registered. Image-map registration develops polynomial functions. To determine the unknown coefficient of these polynomials, well-distributed control points are used. Once the polynomial functions are found, grid location and pixel center mapping is performed; however, grid position is rarely located at the pixel centers requiring the application of resampling techniques. Resampling methods used to estimate the brightness of these pixels can be bi-linear interpolation, cubic convolution, or nearest neighbor. This process is often called geocoding or georeferencing. Image to image registration follows the same steps as map-image registration.

#### Aerial-based remote sensing

Aircrafts, on the other hand, do not produce global images; however, images with high spatial resolution can be obtained. Aircrafts is considered unstable since it moves through the atmospheric zone. The sensors mounted on the aircraft can be reconfigured from a flight-to-flight and the images are captured through a small atmospheric column, unlike their satellite counterparts. Drones are similar to aircraft, but they can fly at low altitudes, which produces images with ultra-high spatial resolution. Drone sensors can also be reconfigured from flight to flight and can capture images through small atmospheric columns. The geometric distortions present in satellite-based remote sensing are most likely present in aerial based remote sensing. UAV-based remote sensing faces several challenges such as the presence of additional sources of image distortions, such as high sensitivity to the wind and jitters due to its lightweight and small volume. Small cameras can cause image distortion because of focal length changes.

Automatic geometric distortion correction must be created for these platforms. The authors of [51] proposed geometric distortion rectification in images captured using UAV, which eliminated rotational error and overlapping regions. The authors of [52, 53] proposed a genetic and neural network to fix the geometric distortion in UAV-based remote sensing. There is also a need to determine optimal flight parameters, such as altitude, speed, number of flights needed to cover the field, and UAV battery life. For instance, if one chooses to fly at 10m or 20m of altitude, how much difference this makes in terms of SCN detection accuracy is one question that can be investigated.

We have gathered some data related to Prosper and Casselton fields to determine optimal flight parameters. We used a multirotor UAV (DJI Matrice M600 Pro with a MicaSense Rededge Mx multispectral sensor) and P-mode flight modes which are GPS position holds. The sensor has a stand-alone GPS antenna that directly geotags the obtained images. We can control the inputs for altitude, sensor overlaps; however, we are limited to a slider bar that adjusts speed from slow to fast with 4 points in between: generally from 1 m/s to 3 m/s based on desired altitude. This application allows us to have full control of the drone, including the ability to stop the mission at any time, order return to land (RTL), or have the drone descend to avoid any conflict with manned aircraft. Table 6 indicates that the total time needed to fly over Prosper field, which has an area of 62 m $\times$ 106 m, was approximately 29 min at an altitude of 10 m, 15 min at an altitude of 15 m, approximately 9 min for an altitude of 20 m, and 4 min when flying at an altitude of 50 m. For Casselton, flying over a field with a total area of 53 m $\times$ 66 m takes approximately 27 min at an altitude of 10 m and 3 min at an altitude of 50 m. The altitude influences the time required to capture images and the total time required to pre-process the collected images. At an altitude of 10 m, 6800 images were collected, and at an altitude of 15 m, 3500 images were collected. More time is needed to stitch these images together and generate vegetation indices at low altitudes, which will allow  us to obtain images with high spatial resolution. The impact of the altitude on detection accuracy and the relevance of certain multispectral bands in determining diseased or healthy crop covers remains unknown and must be studied.

**Table 6:** Altitudes, flight times, and average number dataset size for Prosper and Casselton

Altitude	Time (Prosper field)	Time (Casselton field)	No. of images
10 m	@1.7 m/s = 29 min	@1 m/s = 27 min	$\sim$ 6800
15 m	@2.5 m/s = 25 min	@2 m/s = 8 min	$\sim$ 3500
20 m	@3.3 m/s = 9 min	@2.5 m/s = 7 min	$\sim$ 2900
50 m	@8 m/s = 4 min	@8 m/s = 3 min	–

#### Ground-based remote sensing

Ground-based remote sensing can be used with fixed, hand-held, vehicle mount, and overhead irrigation mount platforms. The use of vehicle and overhead irrigation mounts enable low-cost and high resolution field imagery. Different sensors can be used with these platforms, such as from crioCircle and LIDAR, which are regarded as active sensors since they provide their own light source. Other passive sensors that can be used are visible and near infrared and thermal sensors. Ground-based remote sensing is used for SCN detection. The authors of [54] conducted ground-remote sensing for SCN detection. They used two hand-held and multispectral radiometers (CROPSCAN). The reflected sunlight was measured in the near-infrared region from a high of 3 m above the soil. A variety of sensors are used for remote sensing: data and media. Data sensors capture analog environmental values and convert them to a digital format with the help of deployed sensor nodes in the field. The data captured are collected in the form of multi-hop communications or via a data MULE [55]. Example of data sensors includes NPK, soil moisture, pH, temperature, and humidity sensors. Media sensors collect data in the form of images or video. Most systems use manned or unmanned mobile vehicles for media sensors. The captured media data are transferred to a base station for processing and include hyperspectral, multispectral, and IR cameras. Data sensors are deployed and active at all times to capture PA anomalies occurring in the field. Media sensors, such as hyperspectral cameras, are attached to UAV platforms or aircraft to capture field data while flying. A hyperspectral image is made up of reflections from hundreds of different bands in the electromagnetic spectrum, where each object exhibits a unique reflection characteristic; therefore, similar looking objects with different characteristics can be separated. Multispectral and hyperspectral sensors with high spatial resolution have small footprints. The spatial resolution of the data may not enough to estimate some of the vegetation indexes even though the sensors used in the satellite are capable of sensing a wide area while moving along its trajectory. Temporal resolution defines the frequency at which sensing is completed depending on the platform where the sensor is integrated. The temporal resolution varies and depends on a multitude of factors for different levels of sensing ranging from ground, air, and space.

Table 7 summarizes the three methods of remote sensing in terms of their advantages and disadvantages.

**Table 7:** Precision agriculture sensing types—advantages and limitations

Types	Concept	Advantages	Disadvantages
Satellite-based [56–59]	$\bullet$ Leo-satellite orbit $\bullet$ Sensors configuration fixed	$\bullet$ Earth’s surface image $\bullet$ Low cost $\bullet$ Stable	$\bullet$ Low spacial resolution $\bullet$ Geometric distortions $\bullet$ Complete atmospheric column
Ground-based [60–63]	$\bullet$ fixed,hand-held,vehicle, overhead irrigation Mount sensors $\bullet$ Reconfigurable sensors	$\bullet$ High spatial resolution $\bullet$ Low cost $\bullet$ Narrow atmospheric columns	$\bullet$ Partial/regional earth’s surface $\bullet$ Unstable $\bullet$ Moves through the atmospheric zone
Aerial-based [64–67]	$\bullet$ Sensors mounted on drones/aircraft $\bullet$ Re-configurable sensors	$\bullet$ High resolution $\bullet$ High control $\bullet$ Low cost	$\bullet$ Several flights needed to cover the field

#### Spectral vegetation indices

Vegetation indices (VI) [68] are the variables used to enhance vegetation properties to measure spatial and temporal performance based on the spectral transformation of more than one electromagnetic spectrum band. VI uses spectral wavelengths ranging from 300 nm to 1700 nm, and from ultraviolet, visible light, and near to far infrared spectra for calculations. VIs are mathematical expressions used to measure the reflectance to assist in evaluating crop growth, water index, carbon emission. and similar vegetation properties. Commonly used vegetation indices are listed in Tables 8 and 9. The most popular VIs are NDVI, RDVI, and SAVI. The resultant reflectance map is analyzed to examine the blue and red regions and identify low plant reflectance. The values corresponding to low reflectance indicate abnormal spatial and temporal indications in-terms of plant growth. ‘Type’ specifies the mode of data acquisition in order to calculate the respective VI (please see Fig. 18). It has to be noted that satellites such as Moderate Resolution Imaging Spectroradiometer (MODIS), AVHRR, and Sentinel-2 are simply viable examples for satellites used previously in precision agriculture but may not necessarily be the only options. VI accuracy can be affected by solar position, viewing geometry, land surface, and atmospheric effects [69]. VI applications include:

**Fig. 17:**
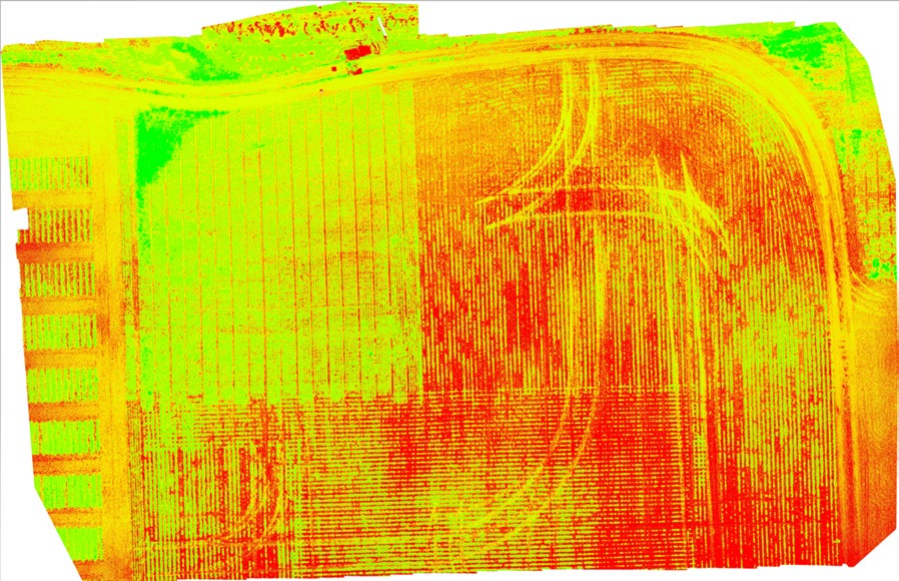
Prosper field June 25th 2021 Alt 20 meters LCI generated using PIX4Dmapper software

**Fig. 18:**
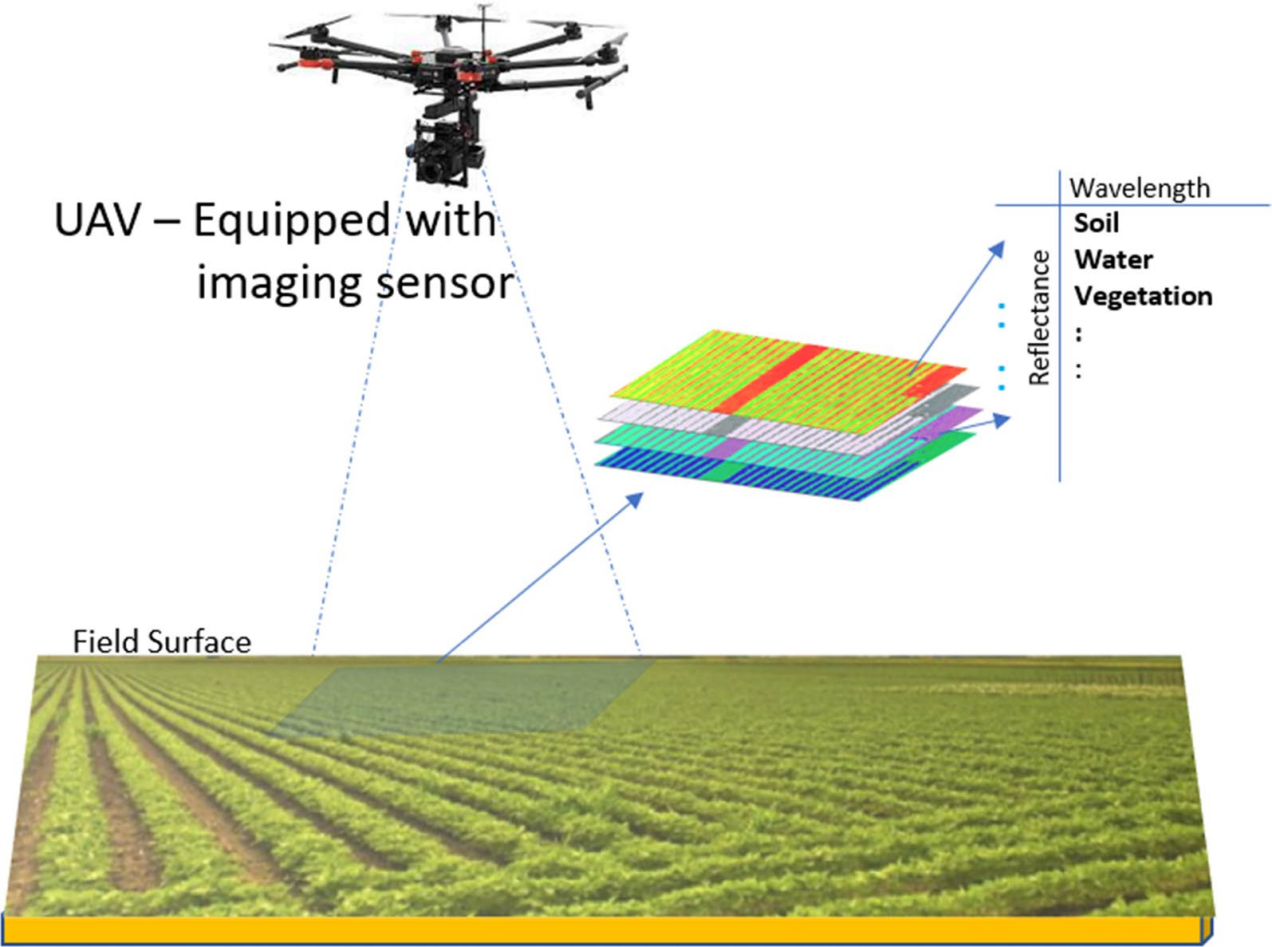
Scenario of UAV based VI calculation [70]

**Fig. 19:**
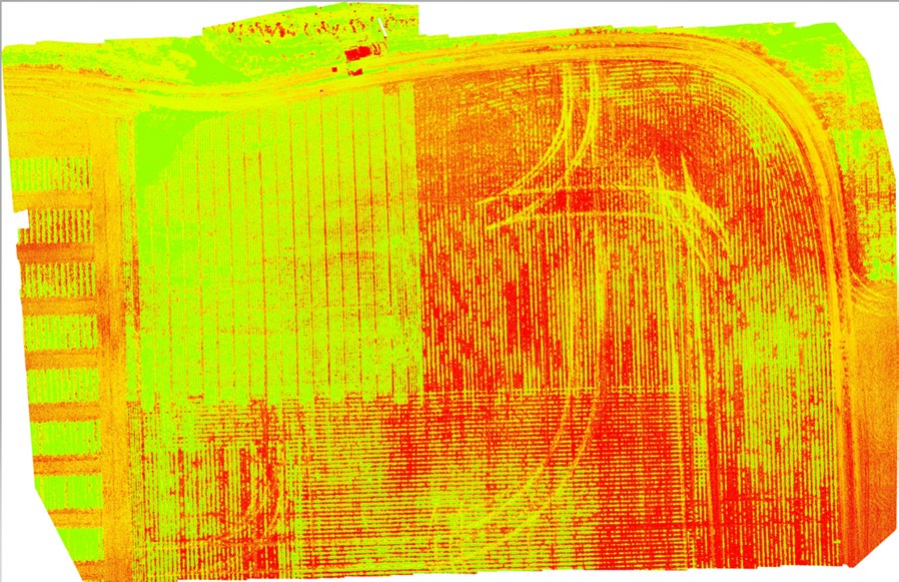
Prosper field June 25th 2021 Alt 20 meters NDVI generated using PIX4Dmapper software

**Table 8:** Vegetation indices for plant yield

Vegetation Index	Equation	Applications	Range (nm)/Type
Atmospheric Resistant Vegetation Index (ARVI) [71, 72]	$\frac{R_{NIR} - R_{RedBlue}}{R_{NIR} + R_{RedBlue}}$	Disease, Weed Mapping	500-900/Satellite (MODIS)
Normalized Difference Vegetation Index (NDVI) [73]	$\frac{R_{NIR} - R_{red}}{R_{NIR} + R_{red}}$	Biomass, Crop Yield	630-900/ Satellite, UAV
Ratio Vegetation Index (RVI)	$\frac{R_{NIR}}{R_{red}}$	Crop Yield, Biomass	630-900/N.A.
Enhanced Vegetation Index (EVI) [72]	$\frac{2.5(R_{NIR} - R_{Red})}{R_{NIR} = 6R_{Red} - 7.5R_{Blue} + 1 }$	Disease, Biomass	500-900/Ground-based, UAV
Re-normalized Difference Vegetation Index (RDVI) [74]	$\frac{R_{NIR} - R_{red}}{\sqrt{R_{NIR} + R_{red}}}$	Crop Yield, Nitrogen Uptake, Soil Moisture, Biomass	630-900/Ground-based
Atmospherically Effect Resistant Vegetation Index (IAVI) [71]	$\frac{R_{NIR} - (R_{red} - \lambda (R_{blue} - R_{red}))}{R_{NIR} + (R_{red} - \lambda (R_{blue} - R_{red}))}$	Crop Yield	500-900/Satellite (MODIS)
Green NDVI (GNDVI)	$\frac{R_{NIR} - R_{green}}{R_{NIR} + R_{green}}$	Water Stress, Crop Yield, Biomass, Disease	520-900/ N.A.
Normalized Difference Red Edge (NDRE)	$\frac{R_{NIR} - R_{rededge}}{R_{NIR} + R_{rededge}}$	Crop Yield, Nitrogen Uptake, disease	700-900/N.A.
Red Edge Difference Vegetation Index (REDVI)	$R_{NIR} - R_{RedEdge}$	Crop Yield, Biomass, Nitrogen Management	700-900/Ground-based (Sprayer)
Red Edge Normalized Difference Vegetation Index (RENDVI) [75]	$\frac{R_{750} - R_{705}}{R_{750} + R_{705}}$	Crop Yield, Irrigation Management, Disease and Nitrogen Management	705-750/Satellite (Sentinel-2)
Wide Dynamic Range VegetationIndex (WDRVI) [76]	$\frac{aR_{NIR} - R_{red}}{aR_{NIR} + R_{red}}$	Nitrogen Application, Crop Yield	630-900/Satellite (AVHRR)
Plant Senescence Reflectance Index (PSRI) [77]	$\frac{R_{680} - R_{550}}{R_{750}}$	Crop Yield, Disease, Biomass	550-750/Satellite (MODIS)
Triangular Vegetation Index (TVI) [78]	$0.5\times (120\times (R_{750} - R_{550}) -$	Disease	550–750/Mount-based,
	$200\times (R_{670} - R_{550}))$		UAV
Red Edge Inflection Point (REIP) [79]	$700 + 40\times \frac{\frac{R_{667} + R_{782}}{2} - R_{702}}{R_{738} - R_{702}}$	Yield and Biomass	667-782/Satellite (MODIS)

**Table 9:** Vegetation indices for nutrients and chlorophyll

Vegetation Index	Equation	Applications	Range (nm)/Type
**Nutrient Management Indexes**			
Soil Adjusted Vegetation Index (SAVI) [80]	$\frac{(R_{NIR} - R_{Red})(1+L)}{R_{NIR} + R_{Red} +L}$	Nitrogen Application, Crop Yield, Disease, Biomass, Water Stress	630-900/UAV
Water Balance Index (WABI) [81]	$\frac{R_{1500} - R_{531}}{R_{1500}+R_{531}}$	Irrigation Scheduling	531 -1500/ Manual Mount-based
Transformed Soil Adjusted Vegetation Index (TSAVI) [82]	$\frac{a(R_{NIR} - aR_{Red} - b)}{R_{Red} + aR_{NIR} - ab}$	Water Stress, Crop Yield	630-900/Manual Mount-based, UAV
Optimized Soil Adjusted Vegetation Index (OSAVI)	$\frac{1.16(R_{NIR} - R_{Red})}{R_{NIR} + R_{Red} + 0.16}$	Crop Yield, Biomass, Nitrogen Management, Soil Moisture, Water Stress	630-900/N.A.
Photochemical Reflectance Index (PRI) [83]	$\frac{R_{531} - R_{570}}{R_{531} + R_{570}}$	Disease, Crop Yield, Leaf Water Stress, Canopy Temperature, Water Stress	531-570/Manual Ground-based
Shortwave Infrared Water Stress Index (SIWSI) [84]	$\frac{R_{858.5} - R_{1640}}{R_{858.5} + R_{1640}}$	Leaf Water Content	858.5-1640/Satellite (MODIS)
Degrees Above Non-stressed Canopy (DANS) [85]	$min(0, T_{stressed} - T_{non-stressed})$	Water Stress	700-1000/ (IR Radiation)/Ground-based
Degrees Above Canopy Threshold (DACT) [86]	$max(0, T_{measured} - T_{ref})$	Water Stress	700-1000/ (IR Radiation)/Ground-based
Normalized Difference Water Index (NDWI) [87]	$\frac{R_{Green} - R_{NIR}}{R_{Green} + R_{NIR}}$	Soil Moisture and Yield	550-900/Satellite (ETM+, SPOT, ASTER, MODIS)
**Chlorophyll Related Indexes**			
Green Chlorophyll Index (GCI) [88]	$\frac{R_{NIR}}{R_{Green}} -1$	Chlorophyll Content	550-900/Manual Mount-based
Normalized Pigment Chlorophyll Index (NPCI) [89]	$\frac{R_{680} - R_{430}}{R_{680} + R_{430}}$	Water Stress through Chlorophyll Content	430-680/Satellite (AVHRR)
Chlorophyll Absorption Ratio Index (CARI) [90]	$\frac{R_{700}}{R_{670}} \times \frac{aR_{670} + bR_{670}}{\sqrt{a^{2}+1}}$	Chlorophyll Content	670-700/Satellite (Hyperion/EO-1)
Chlorophyll Vegetation Index (CVI) [90]	$\frac{R_{NIR}}{R_{Green}} \times \frac{R_{Red}}{R_{Green}}$	Crop Yield, Crop Growth through Chlorophyll Content	550-900/Ground-based
Chlorophyll Index (CI) [91]	$\frac{R_{NIR}}{(R_{RedEdge})} -1$	Chlorophyll and Nitrogen Content	700-900/Satellite (MERIS)

- *Water stress* VIs have been very useful when calculating water stress in plants. Researchers tested 10 different VIs to express canopy water content (CWC), leaf equivalent water thickness (EWT), and live fuel moisture content (LFMC) [92]. green chlorophyll, red-edge normalized ratio (NR), and red edge chlorophyll index were found to be the most sensitive for the 3 parameters [93].
- *Evapotranspiration* Evapotranspiration is an important aspect of the plant life cycle.
- *Soil moisture* Researchers in [94] used remote sensing data from optical and FTIR to compare results from actual field measured data. NDVI and land surface temperature permutations produce temperature vegetation dryness index (TVDI) to assess soil moisture content. Regression analysis and correlation between TVDI and measured soil moisture content indicated a negative coefficient of $r = 0.79$. TVDI was accurate at the experimental settings, reinforcing that hyperspectral image analysis can be used with different VIs to model soil moisture content.
- *Photosynthesis* Multispectral and hyperspectral sensing has provided us with VI methods such as NVDI and SR. The authors of [95] confirm that NDVI is a sensitive indicator of canopy structure and photosynthesis. Researchers selected optimum wavelengths using partial least square, regression and second derivative methods to predict the chlorophyll and carotenoid content in tea leaves from hyperspectral images in [96]. Researchers in [97] used photochemical reflectance index (PRI) with hyperspectral imaging systems from surface optics to detect dynamic photosynthesis correlated changes in reflectance and PRI.
- *Biomass* Researchers are creating methods for using hyperspectral imaging and VIs to calculate biomass, which is an important indicator for monitoring vegetation degradation and productivity. Zhang et al. [98] use hyperspectral imaging for high precision estimation of Khoorchin grassland biomass in China. This research determined a correlation of 0.636 between the normalized difference vegetation index (NDVI) calculated with an NIR hyperspectral spectrometer and a thermatic mapper from a satellite. In [99], researchers indicated that UAV based hyperspectral imaging can be used for the biomass estimation of *Pyropia* (a type of alga) and serves as a cost effective solution for offshore algae monitoring.
- *Disease management* Manual or autonomous field scouting is a convenient method of disease detection. manual scouting is costly due to analysis time, human error, and labor intensity. Diseases that affect soybeans, such as SDS and nematodes, can be detected using remote sensing [100]. Applying ML with computer vision to a hyper spectral image can detect certain diseases in their early stages of development [101]. The use of spectral disease indices (SDIs) to increase disease detection accuracy is common in certain crops [102] and  needs to be investigated since it gives more accurate results for disease detection than NDVI.
- *Weed management* The use of herbicides in weed affected areas is a conventional method of weed management. Remote sensing is used to map the weed spread in the field and report it to farmers for counter action. Spectral images can be used to map the weeds from the crops based on its phenological or morphological attributes. Use of unsupervised ML classification approaches are more accurate than supervised weed detection and mapping [103, 104].
- *Crop Monitoring and yield* There is still a need to investigate remote sensing and ML approaches to improve spatial and temporal issues despite efforts to improve the soybean yield. Biomass, vegetation coverage, plant height, and LAI are essential crop health and development indicators. Remote sensing can obtain information on soil, topography, water management, and various biotic and abiotic stresses.

Figures 17 and 19 indicate examples of vegetation indices of Prosper field from multispectral images captured at 20m generated from a software called Pix4DMapper.

### Machine learning methods for soybean detection

**Fig. 20:**
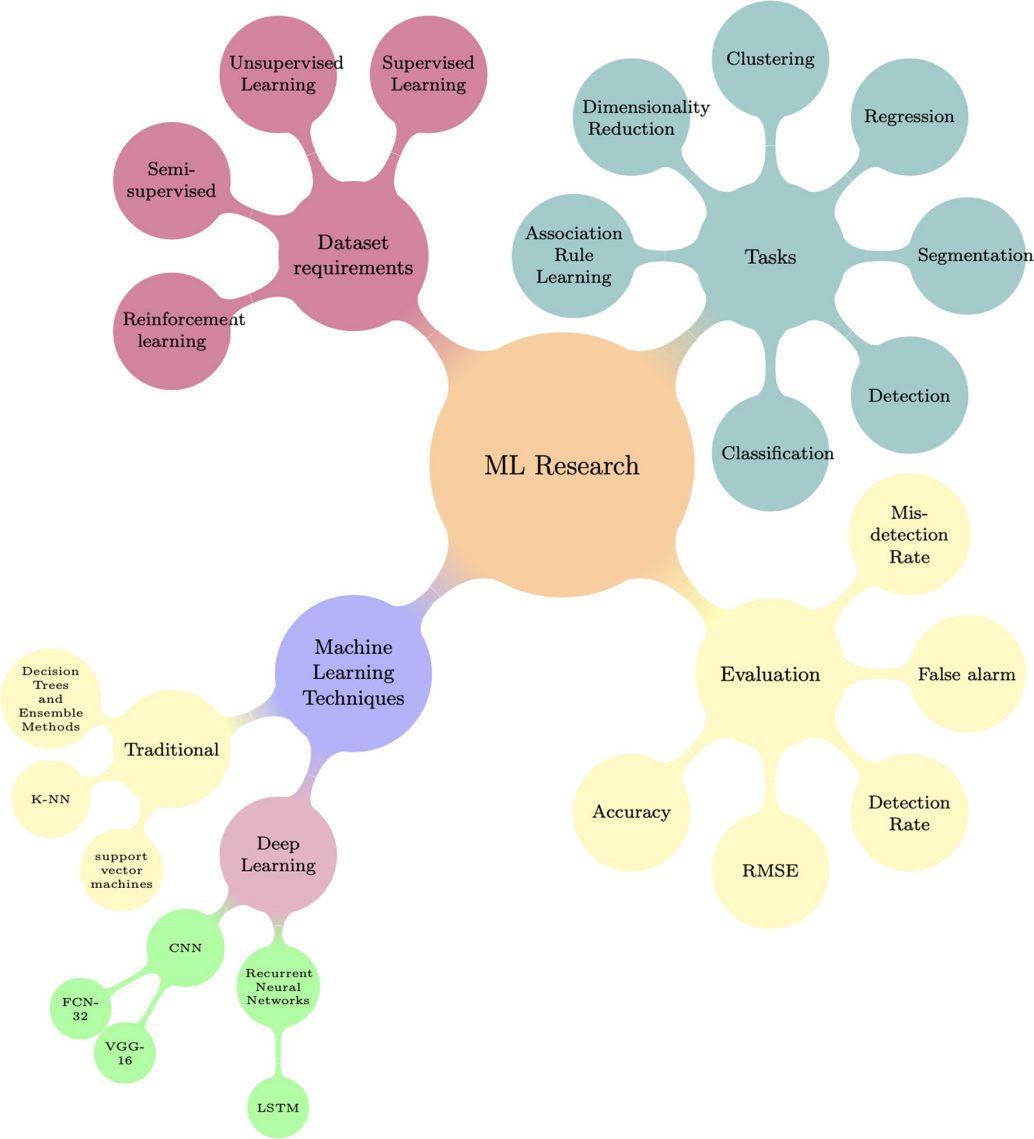
ML research in agriculture

Techniques of machine learning based SCN detection can be grouped into two sub-categories: supervised ML and unsupervised ML. Supervised ML can be grouped into machine learning based and deep learning based (see Fig. 20). ML based SCN detection includes linear regression, support vector machine, neural network, decision trees, and ensemble methods such as random forest.

Deep learning based SCN detection techniques include deep neural and convolutional neural networks. Machine learning techniques require feature selection, and several models can be used based on the desired output. Features such as weather data, soil properties, and locations can be used as inputs for a machine learning classifier.

Machine learning and deep learning algorithms can be categorized into supervised and unsupervised. Supervised algorithms require a labeled dataset. The dataset consists of hundreds of images with labels: healthy soybean or infested soybean. The algorithms learns the correlations and patterns between the input features extracted from the image and the output. Labeling datasets for SCN detection is challenging since it requires obtaining soil samples and associating them with the images of earth’s surface. Hyperspectral features extracted from hyperspectral images can be labeled as “healthy soybean” or “infested soybean” classes using soybean samples. This dataset is then used to train supervised learning techniques such as random forest, linear and logistic regression, and decision trees. Unsupervised algorithms do not require labeled datasets, they attempt to map each sample to one class. Examples of unsupervised algorithms include kNN, K-means, neural networks, and principal component analysis (PCA).

#### Logistic regression

Logistic regression is a simple classifier technique that has been used for SCN detection [54] and remote sensing in general. This technique uses sigmoid functions to determine the classifier outputs. The output of the classification is either 0 (no-SCN detected) and 1 (SCN present) in binary classification. Linear regression uses an input, which can be a vector of features, and computes the output using a sigmoid function. If the output is higher than a certain threshold, linear regression outputs 0, or otherwise outputs 1.

#### Decision trees and ensemble methods

Decision trees are supervised ML techniques that provide simplicity and a high level of interpretability [105]. We can distinguish between several decision tree algorithms, such as CART, C4.5, ID3, and CHAID. ID3, proposed in 1986, builds the decision tree using information gain (IG) and entropy with nominal features. The root is the nominal attributes whose gain is the highest. ID3-based decision tree models are simple and fast but are prone to over-fitting, which was overcome using the gain ratio in 1993, when C4.5 was proposed. C4.5 splits attributes based on a threshold, which is selected to maximize attribute gain. CART is based on a GINI index and works with nominal features. CHAID performs the Chi-square test to determine the significant attribute.

The authors of [106] proposed a decision tree using entropy and Gini-index to classify soybean crop diseases. The authors used a UCI machine learning dataset consisting of 307 samples, 35 attributes, and 19 classes including soybean cyst nematodes. The dataset was split into 80% training and 20% testing. The reported results indicated that entropy decision tree was 80.6% accurate, and the gini-index decision tree was 82% accurate. Bhatia and colleagues [107] proposed an enhanced Decision Tree Classifier (DTC) trained on the SoyBean Large (SBL) dataset from the UC Irvine Machine Learning Repository to predict 19 soybean diseases, or labels. This model was introduced to improve upon variations of the existing Classification and Regression Tree (CART) algorithms using a DT algorithm, a feature selection technique, and Random Over (RO) sampling. The SBL dataset contained 35 features that were used to identify the best feature subsets by applying three feature selection techniques: Correlation-based Feature Selection (CFS), Random Forest Importance (RFI), and the cons filter. Eight common features that were deemed relevant were chosen by these three techniques: precipitation, temperature, leaf spot size, incidence time, leaf mildew growth, area damage, canker lesion color, and fruits spot. Results indicated that the enhanced DTC fitted with RFI, CFS, and cons filter achieved performance greater than 93% when using accuracy  and AUC metrics. The highest performing model using the enhanced DTC was a C4.5 DT algorithm with an RFI filter, producing an accuracy  of 98.1%.

However, the decision tree method has various disadvantages, such as high variance; therefore, ensemble techniques have been proposed to reduce the high variance of this method. Ensemble methods are defined as techniques that uses multiple decision trees to enhance the performance of the model. The way these trees are combined varies based on the task (classification or regression) and the type of the ensemble methods (bagging, random forest, and boosting techniques). For instance, if we deal with classification problem such as (SCN detection), then the majority vote is considered. If we deal with regression problem, such as predictig soybean yield or price, then the average of the outputs is considered.

Bagging classifiers use each subset of the original dataset and aggregate the individual predictions to derive the final prediction [108]. Bagging techniques enhances the performance of the classifier. For instance, the authors of [106] indicated that bagging decision trees can improve the soybean cyst nematodes classification accuracy by 8% compared to entropy decision trees. However, bagging often creates correlated trees, which does not result in a reduction of high variance. To overcome this issue, random forest  has been proposed.

Random forest  is a popular ensemble machine learning algorithm used for classification and regression [109] especially in situations where the datasets have high-dimensionality [110]. Random Forest attempts to fix one of the fundamental decision tree problems: overfitting. Random forest is built by grouping several decision trees, similar to bagging ensemble methods. The features selected in the first subset are exclusively used with this current subset and are not selected in the next subset; therefore, random forest is considered as bagging generalization. The algorithm attempts to solve this issue by creating a forest, or several decision trees, using a meta-algorithm called Bootstrap Aggregation, or Bagging. This algorithm utilizes a subset of features and records to train each tree within the forest in parallel. Random forest is robust enough to achieve high accuracy with very little feature engineering due to the special implementation of bagging. A higher number of trees utilized to create the model dictates the accuracy of the results in real time. The input data is fed to each tree in parallel and each tree makes its prediction based on the subset of features and records it was trained on. A voting scheme is used to determine the prediction with the greatest number of votes. The authors of [111] proposed a random forest for distinguishing soybean varieties from weeds, Palmer amaranth and redroot pigweed, that cause yield reductions in the US. Random forest was used with leaf multispectral reflectance data for this classification.

Boosting is another form of ensemble method where the trees grow sequentially [112]. This approach begins with a weak classifier and gradually uses it to create a stronger classifier. It starts with node tree, which contains all the observations [113]. The residuals calculated from the predictions obtained from the previous tree are then used to fit the next tree, which adjusts the residuals by multiplying the tree with a shrinking parameter before it can be added to the original tree. This process is repeated until it converges with the optimal decision tree. Gradient boosting learns slowly as it builds the stronger model from a small tree. This method uses the number of trees, the shrinkage parameter, and the boosting tree depth as inputs. Gradient boosting is prone to over-fitting if many trees are used. XG boost augments gradient boosting using a regularization parameter to control the over-fitting. This model is also known as regularized gradient boosting. Adaptive boosting [114], like gradient boosting, also begins with one tree. This method weighs the mis-classified observations and retrains the model after accounting for these weights to build a new decision tree. This process is repeated until convergence. This method is a form of boosting because the tree at each iteration is built based on the tree of the previous iteration. A performance comparison between random forest, gradient boosting machine, XGBoost, SVR, MLP, and KNN for agribusiness forecasting is proposed [115]. According to this study, random forest outperforms the other algorithms.

#### Support vector machine (SVM)

Support vector machine (SVM) is a machine learning method that attempts to segment data points by creating one or more hyperplanes in high-dimensional space [116]. Data that are separated by the hyperplane(s) can be considered clusters, the detection of nutrition. SVM has historically been utilized to analyze plant nutrient deficiency, and crop and weed classification with high levels of accuracy, using detection of nutrition and machine learning approaches. Kernels can be applied to improve performance and achieve good separation on non-linear or high-dimensional space. Kernel functions include linear, radial, sigmoid, and polynomial [116]. Training SVM models consists of finding the hyper-parameters of the kernel that maximize the separation between the two classes [112] by minimizing the cost function. SVM was originally developed for classification and modified for regression tasks(called Support Vector Regressor (SVR)) for short term soil analysis.

The authors of [45] proposed soybean variety classification based on hyperspectral features extracted from hyperspectral images then fed them to a linear support vector machine (LSTM). This model was trained to distinguish between 35 different soybean varieties. The authors of [117] proposed the use of a support vector machine with different kernels for plant discrimination based on NDVIs. The authors of [118] developed the use of a deep support vector machine (DSVM) for hyperspectral image classification. The authors classified indian pines including soybean-mintill, soybean-clean, and soybean-notill, using hyperspectral images with an accuracy of 100% for soybean-mintill, soybean-clean, and soybean-notill. The authors of [112] proposed machine learning methods including SVM for charcoal rot prediction in soybean. The authors of [2] proposed the use of a support vector machine for US crop type classification: corn, cotton, rice, soybean, and winter wheat. The authors of [119] highlighted the need for a fully automated framework to process big data that resulted in the high-throughput phenotyping. The authors identified several research gaps that exist when using HTP. Most of research related to phenotyping is limited to a set of machine learning tools, such as support vector machine and artificial neural networks, while there are several recent advances in the field of machine learning and deep learning that are far more efficient.

#### Principal components transformation 

Principal components transformation is a transformation applied on original hyperspectral bands, which are correlated to define new bands where no correlation exists in the spectral space. This transformation is one of many techniques, such as wavelet transform and Fourier transform, and is used for dimensionality reduction. This method uses a vector of bands and multiplies them with a transformation matrix to obtain new bands. The transformation matrix is derived from the covariance matrix. When the singular-value decomposition is performed, the transformation matrix is the transposed matrix of the eigenvectors from the diagonal form of the covariance matrix. Once we determine the eigenvalues, we can form the transformation matrix which transforms the hyperspectral bands’ vector into new bands.

#### k-Nearest neighbor (kNN)

k-Nearest neighbor (kNN) is one of the oldest, most popular machine learning algorithms used today [120, 121]. This algorithm is a special type of machine learning called an unsupervised model, often used for classification problems [120]. The algorithm attempts to label unlabeled data points by selecting the majority label based on k neighboring points in the training data [121]. Points are considered neighbors based on euclidean distance. The kNN algorithm is simple, easy to understand, and implement due to its relatively simple algorithm. kNN has been used for soybean disease classification. For instance, the authors of [122] proposed kNN for disease classification and compared its performance to SVM, neural network, Naive Bayes, and decision tree.

#### Deep learning and convolutional neural networks

The neural network is one of the main innovations in machine learning, which is an algorithm based on the fundamental understanding of biological neural network (BNN) structures. This network is built using logistic regression bricks that consist of neurons with one activation function, such as sigmoid function or softmax. Neural networks rely on two concepts: the input is fed-forward to calculate the target and the error is back-propagated between the computed target and the actual target. Training neural network consists of finding the optimal weights $w_{ij}^{l}$. The intensity of change is scaled using the learning rate. A higher learning rate makes more dramatic changes during the adjustment process while a small learning rate has the inverse effect. This backpropagation method ultimately attempts to minimize the loss and maximize predictive power and accuracy. Neural network is a powerful classifier; therefore, it has been proposed for soybean crop disease classification. The authors of [106] proposed a neural network to classify soybean cyst nematodes from other diseases in the crop. Neural networks have been applied to soybean yield predictions. The authors of [123] proposed a neural network to correlate soybean yield to topography, soil fertility, weather conditions, and evaluate the artificial neural network's (ANN) ability to attribute yield loss due to SCN.

#### CNN-based SCN detection

Convolutional neural network, CNN, builds upon convolution layers and fully connected layers. Convolutional layers aims to reduce the input feature size with successive convolutions. Hyperspectral images require features extraction, which is a tedious process with traditional machine learning techniques. CNN provides automatic feature extraction and performs better than PCA.

There are three main tasks used to build convolution layers: convolution, rectified linear unit and pooling.

**Fig. 21:**
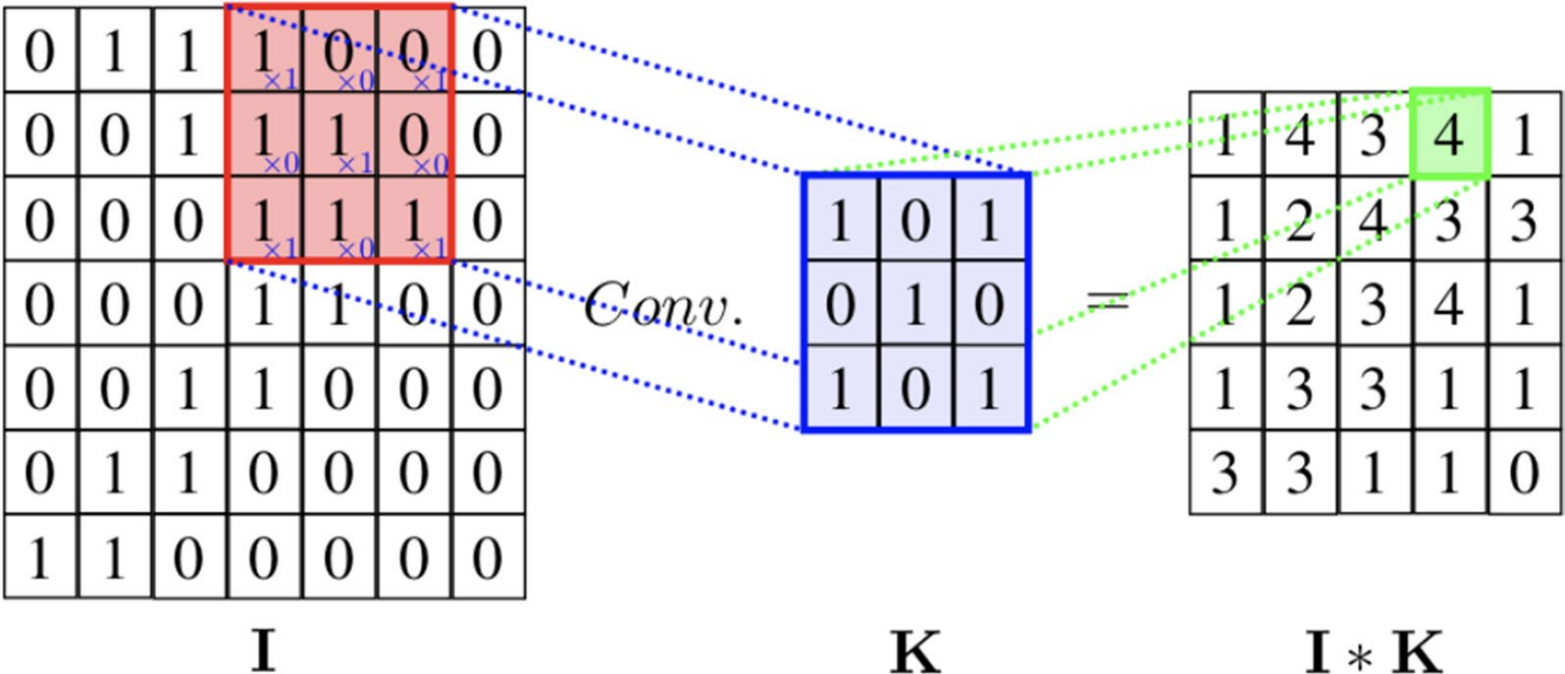
Convolution operation

*Convolution* Convolution layers perform automatic feature extraction and reduce the dimensionality of the input image. The input raw images goes through a set of convolutional filters. This operation is described in Fig. 21 where the filter *K* is of size $3\times 3$ and the input image is of size $7 \times 7$. The filter is slided through the image and each time, it outputs the result of the convolution between the filter and the sub-matrix of the input image. At the end of the operation, we end up with a matrix of size $5\times 5$.

*Activation* allows for faster and more effective training by mapping negative values to zero and maintaining only the positive values. This method is sometimes referred to as activation because only the activated features are carried forward into the next layers.

*Pooling* simplifies the output of the convolution operation by performing nonlinear downsampling. Other types of pooling can be used such as the minimum and average pooling. This operation reduces the number of parameters fed to the neural network and solve the issue of the curse of dimensionality. This is advantageous because it reduces overfitting and training time by reducing the size of the neural network.

CNN is often used with a neural network as a classifier; however, CNN can be used with other machine learning classifiers, such as random forest or support vector machine. We distinguish between several CNN architectures based on how many convolution layers the architecture has, the type of the pooling used, and how many fully connected layers are used.

The authors of [124] proposed a CNN for soybean nematodes detection using microscopic images. The authors investigated several architectures such as Xception, VGG16, InceptionV3, ResNet50, ResNet101, InceptionResNetV2, DenseNet121, DenseNet196, DenseNet201, and EfficientNetBx and achieved an accuracy of 96%. Akontaya et al. [125] proposed a “Convolutional Selective Autoencoder” (CSAE). This supervised machine learning paradigm involves a dataset that was composed of 644 microscopic soil sample images. These images were then SCN marked by nematologists using a Matlab-based mobile application and pre-processed from a 3-channel RGB to a 2-channel grayscale image whose pixel values were normalized. Images were classified according to “high-cluttered” and “less-cluttered” groups that signifies a higher SCN egg density alongside soil debris or a lower density of SCN eggs respectively. The machine learning model was a trained and tested with a 80/20 split and a learning rate CNN of 0.002. This model had an average detection of 95%. The authors of [126] proposed a pixel-wise convolutional neural network trained on datasets collected using near infrared hyperspectral imaging technology to classify three varieties of soybeans (Zhonghuang37, Zhonghuang41, and Zhonghuang55) with an accuracy of 90%. The authors considered pixel-wise spectra, which provides a much larger data volumes than an average spectra of samples to address the large amount of data required for deep learning techniques. The authors of [127] proposed an automatic SCN-eggs count using microscopic images and convolutional neural networks. The authors generated a dataset by collecting random samples from various farms in Iowa with different SCN different infestation levels to train this model. A 1-inch-diameter probe was used to collect soil samples during the Fall of 2015. The authors of [128] proposed ML for soybean plant breeding. The authors of [129] proposed 3D CNN for plant disease detection. The authors of [130] proposed a 3D CNN model with hyperspectral imaging to detect charcoal rot, which is another important plant disease.

#### LSTM-based SCN detection

**Fig. 22:**
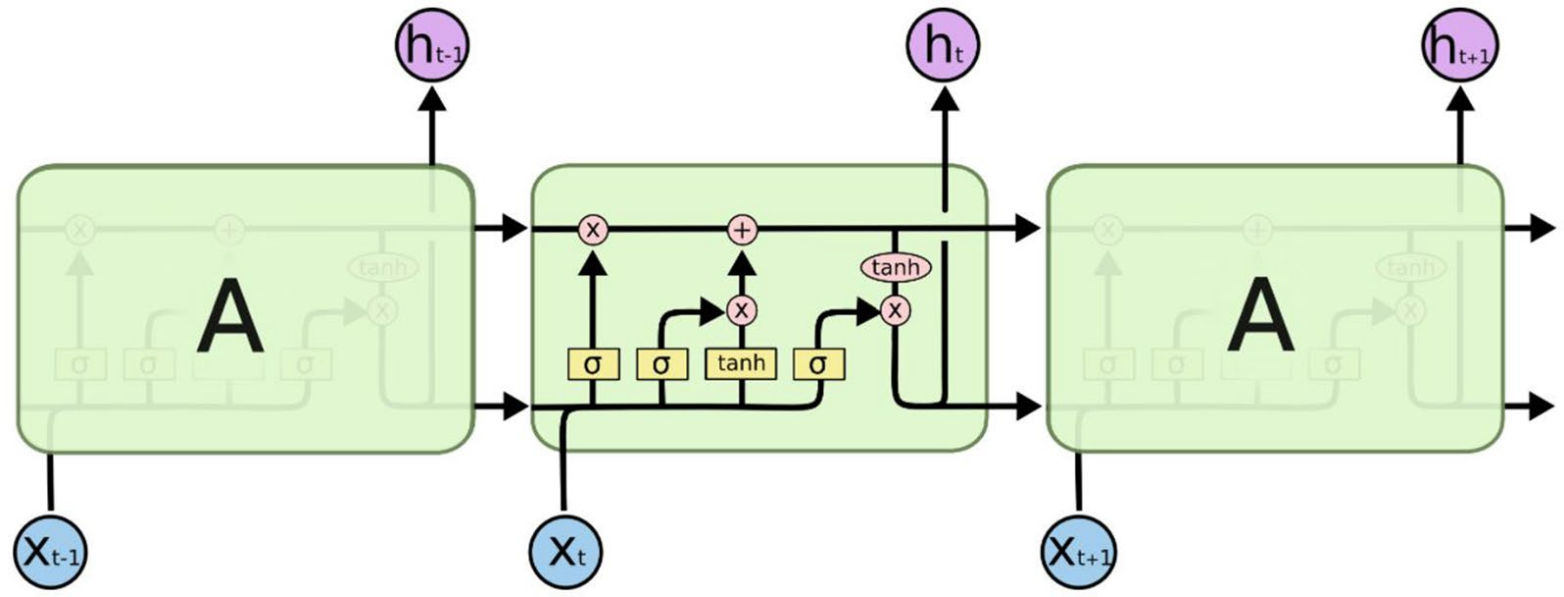
LSTM contains four interacting layers [134]

LSTM is a type of recurrent neural network that has some advantages over neural networks, since neural networks have some limitations when it comes to sequential data. LSTM consists of a loop that connects the input layer to the output layer. The use of this loop enables LSTM to pass information on to perform present tasks. LSTM networks are designed to avoid long-term dependency problems, have been applied to solve many problems related to soybean agriculture, and  to forecast yields in [131–133]. Figure 22 illustrates the typical architecture of LSTM with three interacting layers.

**Table 10:** Confusion matrix

Actual\Predicted	Positive (1)	Negative (0)
Positive(1)	TP	TN
Negative(0)	FP	FN

**Table 11:** Metrics for SCN classification

Classification evaluation metric	Importance
Detection rate	Early prediction of nematodes infestation is a key factor to reduce the disease spread in soybean. High detection rate is desired.
False alarm rate	Models with low false alarm rate can prevent farmers from spending money on mitigation techniques or cultivate soybean especially that its price keeps increasing.
Miss-detection rate	Miss detection of nematodes in soybean can be costly as farmers can experience yield loss and will detect nematodes infestation
Accuracy	This metric combines the previous metrics and higher values of accuracy is required

Metrics such as mean squared error (MSE), root mean square error (RMSE), root mean square relative error (RMSRE), mean absolute error (MAE), mean absolute percentage error (MAPE), and $R^2$ can be used to evaluate regression tasks in machine learning. Tabular visualizations can also done using confusion matrices (see Table 10). Here, the rows represent the actual (or ground truth labels) while the columns show the predicted labels where true positive (TP), true negative (TN), false positive (FP), and false negative (FN) scores can be used to evaluate metrics such as precision, recall, and F-measure but this is beyond the scope of this manuscript. Table 11 shows the 4 commonly used metrics for SCN classification.

Tuning the neural network or decision tree hyperparameter often leads to overestimation bias or high variance; therefore, it is important to evaluate the model. Learning curves are used to determine if the model over-fits or under-fits the dataset. Dataset addition or regularization techniques can be used to control these issues.

### Other machine learning techniques for SCN detection

The authors of [125] proposed a workflow for determining egg count using computer vision and microscopic imaging to overcome the shortcomings of direct methods. This workflow consists of data collection, sampling, and deep learning-based egg counting. The proposed workflow demonstrates the effectiveness of deep learning; it achieves near-human accuracies of 95% on average, with a 93.73% precision score and an F1 score of 0.944.

The authors of [100] proposed a detection methodology based on linear discriminant analysis (LDA), logistic discriminant analysis (LgDA), and linear correlation analysis, and applied to data collected from Boulder, Colorado. These authors collected data weekly, between 11 am and 2pm, for a period of 71 days after planting. The authors reported a 97% accuracy for detecting healthy plants and a 58% accuracy for detecting infested plants. The authors also investigated the correlation between disease rating and selected vegetation indices. The highest correlation reported was greater than 0.8 between the disease rating and VI occurred during 112 days after planting (DAP).

The authors of [135] investigated the classification of normal from insect-damaged vegetables in soybeans considering 100 vegetable soybean pods. The authors used hyperspectral imaging with spectrum of 400 nm and 1000 nm, extracting features such as min, max, mean, and standard deviation, and feeding them to the SVDD classifier with an accuracy of 97.3% for healthy plants and 87.5% for insect-damaged plants. Sucrose centrifugation is the most common technique used to separate debris from extracted nematode egg suspensions. The authors of [136] proposed a new method,“OptiPrep”, to improve separation and recover extracted eggs. They also proposed a machine learning based automatic egg count. The authors of [54] investigated the use of remote sensing coupled with geographic information system (GIS) technologies to create new tools for detecting and quantifying SCN population densities and their impact on yields. The authors obtained Landsat 7 satellite images of a field near AMES, Iowa for five days during the 2000 season. Aerial images were obtained for 12 dates and at a high ranging from 45 m to 425 m. This image collection was accomplished by filtering the reflectance of a near-infrared region of 810 nm. GIS software was used to depict the SCN population densities. The authors of [137] proposed spectral imaging with ensemble machine learning to detect soybean seeds. Ensemble classifiers such as random subspace linear discriminant (RSLD), linear discrimination (LD), and linear support vector machine (LSVM) methods were used to classify soybean varieties. The authors report that the RSLD algorithm had greater stability and reliability compared to LD and LSVM, achieving the highest soybean classification accuracy in 10, 15, 20, and 25 categories. The authors considered 155 features and 15 types of soybeans, with accuracies of 99.2% for RSLD, 98.6% for LD, and 69.7% for LSVM. The authors of [138] review of machine learning spectral imaging for the automatic discernment of crops and weeds as shown in Table 12.

**Table 12:** Machine learning for soybean disease detection

Model	Objective	Dataset	Technology	Collection period	Spectrum range	Performance
CNN [127]	Identifying SCN Egg Count	Soil Samples	Microscopic Imaging	Fall 2015	N/A	ADA(94.33%), AMER(18.18%), AND(99.7%)
GIS + RS [54]	Identifying SCN	Soybean Field near Ames, Iowa, in 2000	Satellite Images and Aerial Images	5 Collection Dates	810 nm	Aerial Images (80%), Satellite Images (47%)
SVDD [135]	Identifying Insect Damage	100 Soybean Samples Harvested from a Garden in Zhejiang Province, China	Hyperspectral Imaging	2011 Harvest Season	400–1000 nm	97.3% (normal), 87.5% (Insect-damaged)
Ensemble ML [137]	Differentiating soybeans Seeds	462 bands (25 varieties and 50 Seeds for Each) 1250 spectral curves	Hyperspectral imaging	N/A	400–1000 nm	RSLD (99.2%), LD (98.6%), LSTM (69.7%)
CNN [125]	Identifying SCN Egg count	Data collected from two fields in the State of Iowa	Microscopic images	Fall 2015 and Spring 2016	N/A	Accuracy (95%), Average precision (93.73), F1-score (0.944)
LDA, LgDA, and LCA [100]	Identifying SCN & SDS	Data collected (800 Leaf Spectra) from (Analytical spectral devices, Boulder, CO, USA)	Spectroscopic analysis in the NIR Region	Weekly basis, 71 days after planting (DAP)	350–1070 nm	97% (Healthy Plants) and 58% (Infested Plants)%
Pixel-wise CNN [126]	Differentiating soybeans seeds	3 Varieties of soybeans were prepared with 1890 soybeans in each variety	Hyperspectral imaging	2019	975–1646 nm	Average accuracy (86%)

### Machine learning for crop yield forcasting

Breeding for yield is a highly complex and non-linear process due to genetic and environmental factors. Crop yield forecasting is important for management as well as providing timely information for optimum management of growing soybean crops and rapid decision-making, better policy making regarding import and export to strengthen national food security. Furthermore, machine learning and deep learning provide accurate predictions and often outperform statistical models. All these factors motivated researchers to use learning techniques for pre-season yield predictions. As a results, several studies have considered machine learning using several models, such as neural network, LSTM, random forest, and kNN (see Table 13) trained on datasets that combine soil properties and weather data obtained from MODIS and weather station as well as some vegetation indexes such as NDVI, and land surface temperature. These models were evaluated using regression metrics such as R2, RMSE, MAE, and MAPE. The authors of [132] proposed a satellite-based machine learning technique with weather data for soybean field forecasts. This study presented a model to perform in-season soybean yield forecasts using Long-Short Term Memory (LSTM), neural networks, satellite imagery, and weather data. The authors compared the performance of three algorithms: LSTM, linear regression, and random forest for forecasting soybean yield using VIs. The model was evaluated using MAE. The authors [139] proposed a model for forecasting US corn and soybean yields using remote sensing variables. The normalized NDVI was derived from MODIS day and nighttime land surface temperature (LST), and weather data from the crop growing seasons of 2006–2011. The authors of [128] investigated several machine learning models, such as support vector machine, random forest, and multi-layer perception, for predicting soybean yields using hyperspectral reflectance, which was collected from two different growth stages on 250 soybean genotypes grown in four different environments. The hyperspectral reflectance range was (395–1005 nm). The R5 growth stage provides more information to predict soybean seed yields., indicating that the RF algorithm achieves the highest performance, with a 84% yield classification accuracy. The authors of [133] proposed a pre-season agriculture yield forecast based on machine learning. The authors used scalable machine learning to perform the forecast using weather and soil properties. A deep neural network used LSTM recurrent layers and four fully connected layers in this model, using soil features such as soil ph, point longitude, point latitude, soil organic carbon content, bulk density, sand content, silt content, and clay content. The authors evaluated the model on US and Brazil soybeans using several evaluation methods such as MAE, MAPE, RMSE, RMSRE, and R2. The authors of [131] investigated the use of a deep convolutional neural network-based LSTM model to predict soybean yield. This model was trained on weather data, MODIS Land Surface Temperature data, and MODIS Surface Reflectance (SR) data, labeled using historical data.

The authors of [140] proposed a Glycine max yield prediction based on deep learning and data fusion with different sensors. They collected RGB, multi-spectral, and thermal images using UAV from Columbia, Missouri. The authors extracted features such as canopy spectral, structure, thermal, and texture features to forecast grain yield using Partial Least Squares Regression (PLSR), Random Forest Regression (RFR), Support Vector Regression (SVR), input-level feature fusion based DNN (DNN-F1), and intermediate-level feature fusion based DNN (DNN-F2).

**Table 13:** Machine learning for crop yield forecasting

References	Crop type	ML algorithm	Features	Evaluation parameter
[131]	Soybean (Glycine Max)	Deep CNN-LSTM	MODIS (LS, and SR) Weather datam	Avg RMSE
[128]	Soybean (Glycine Max)	SVM, RF, and MLP	Spectral reflectance bands	RF (84%)
[132]	Soybean (Glycine Max)	LSTM, LR, Random forest	NDVI, EVI, Land surface temperature	Mean absolute error
[139]	Soybean yields and corn	Regression trees	NDVI, precipitation, LST	$R^2$, RMSE
[133]	Soybean yields and corn	Scalable ML (DNN-LSTM)	NDVI, Precipitation, LST	$R^2$, RMSE, MAE, and MAPE
[140]	Soybean (Glycine Max)	PLSR, SVR, DNN-F1, DNN-F2	Canopy spectral, structure, thermal and texture features	$R^2$ of 0.720 and a (RMSE%) of 15.9%

## SCN management: influence of fertilizers/pesticides/herbicides on crop quality

Experimental data are controversial and there is no general agreement on the impacts of nitrogen fertilization. Fertilizer application, particularly nitrogen, continues to be a controversial topic since soybean production is responsive to N fertilization. Nitrogen application results in a yield increase when applied during the reproductive stage but can also decrease production. Field experiments were conducted in seven Alabama locations to study the response of N fertilization on various growth states [141]. The results indicated that five of seven locations had a positive response to nitrogen fertilization. The response from these five yields were inconsistent with respect to rate and timing. The authors explained this by concluding that the yield’s response to nitrogen application depends on soil nitrate concentration at planting. The authors of [141] reported a yield decrease in response to nitrogen application while a study by the University of Minnesota showed that the application of nitrogen for in-season soybean crops does not have any effect on yield [142] (please see Table 14).

### Fertilizers

**Table 14:** Sources of nitrogen in fertilizer and yield output

Nitrogen source	Timing	Method	Yield
None	–	–	52.4 (bu/acre)
Ammonium sulfate	Pre-plant	Broadcast	54.2
Ammonium sulfate	Early	Broadcast	54.3
Ammonium sulfate	Eary	Knife	52.5
Ammonium sulfate	Pod fill	Broadcast	53.2
Urea	Early	Knife	51.5
Urea	Pod fill	Broadcast	52.4

The authors of [143] studied the effects of soil tillage systems, seeding rate, fertilizer dosage, and time of application on soybean yield and quality over a period of three years. This work analyzed data using statistical approaches, such as ANOVA PoliFact Soft and least significant difference, indicating that nitrogen-46 fertilization during phases V3–V5 had a significant positive quantity effect on soybean production. The authors of [21] reported that Iowa soybean yield loss was by SCN on resistant, PI 88788, will increase as SCN population virulence increases. The authors of [144] proposed the use of convolutional neural network to classify different soybean diseases using visual leaf images. This approach is sound; however, some soybean fields that are infested with SCN do not have any visual symptoms. Soybean is often rotated with corn to manage SCN infestation for this reason; however, there is a lack of knowledge on the mechanisms responsible for SCN population reductions, such as the number of rotation years necessary for effective SCN management [145].

Determining the optimal soybean variety, herbicide, pesticide, and fertilizer combination to maximize yield is difficult with so many options available. Soil conditions and SCN genetics can be extremely diverse and localized within small areas, further complicating selection. However, it seems that there are several general techniques that are likely to improve yield. Soybean varieties that are both SCN and glyphosate-resistant usually increase yield, and the glyphosate-resistant gene does not make the plant more vulnerable to SCN, but may have a minor impact on biological nitrogen fixation. The use of glyphosate will increase yield by eliminating weeds. For pesticides, Clavaria pn should be effective in managing SCN and fluopryam will treat SDS and may reduce SCN population in a limited capacity, but more research is needed. Early application of ammonium sulfate using a broadcast method may also increase yield, and P fertilizer will likely have the same effect.

### Herbicides

#### Glyphosate

Glyphosate is one of the most commonly used agricultural herbicides for soybean and corn fields. This herbicide disrupts the shikimic acid pathway through the inhibition of the the enzyme 5-enolpyruvylshikimate3-phosphate (EPSP) synthase. The resultant deficiency in EPSP production leads to reductions in the aromatic amino acids that are vital for protein synthesis and plant growth [146]. Soybeans have been genetically engineered to be glyphosate resistant or glyphosate tolerant.

A field crops study [147] analyzed the relationship between soybeans and the glyphosate-resistant gene and herbicides, where they monitored biological nitrogen fixation and yield. The authors determined that the gene negatively impacted some aspects of biological nitrogen fixation but had no significant impact on yield over a period of three years. Glyphosate increased the yield during the same period at some locations. These results were not consistent; therefore, biological nitrogen fixation and yield may depend more on location than weed control strategy [147].

Weed technology investigated the relationship between glyphosate-resistant soybeans and SCN in controlled greenhouse conditions because field observations led researchers to believe that there was an interaction between glyphosate and SCN. Completion of this research and subsequent findings did not support these observations, indicating that glyphosate resistance was not compromised by SCN [148].

#### Dicamba

Dicamba is an herbicide used to kill broadleaf weeds by directly eliminating them and to prevent new weed growth. Dicamba is effective at most growth stages, making it an extremely versatile herbicide. This herbicide is absorbed through the roots, leaves, and stems of the plant. Dicamba then imitates auxins, or plant hormones, and causes a wide variety of problems, leading to plant death [149]. Dicamba has the unfortunate side effect of damaging crops due to its propensity to drift onto unintended targets as vapor. One study indicated that this vapor drift can cause as much as a 10% reduction in soybean yield due to detrimental plant effects [150]. Dicamba-tolerant soybean varieties have been created, each with their own problems and benefits [151]. It is difficult to study the effects of these traits on SCN because all dicamba-tolerant strains are also glyphosate-tolerant [152]. The relationship between the dicamba-tolerant gene and SCN is unclear due to these reasons. Another consideration is the impact of dicamba on the soil since it biodegrades fairly quickly under aerobic conditions; therefore, groundwater contamination is improbable [153].

### Pesticides

#### Clavaria

Clavaria pn is a seed treatment method that can manage SCN by reducing its ability to reproduce. The mechanism focuses on the bacteria Pasteuria nishizawae. The bacteria spores protect the roots of the plant by inhabiting the surrounding soil and reduce SCN feeding and reproduction abilities. The spores return to the soil and continue protecting the plant after the nematodes die and decompose. Syngenta, the manufacturer of Clavaria pn, claims that the treatment is effective under variable environmental conditions [154]. An independent study by Iowa State examined the difference in yield between Clavaria and a few other pesticides. SCN soil population density data was collected while the differences were analyzed. The data indicated that Clavaria use correlated with “significant reductions in season-long SCN reproduction.” A consistent and substantial increase in yield was not observed despite a decrease in SCN reproduction [155].

#### Fluopyram

Fluopyram (commonly called ILeVO) is a chemical fungicide used to treat SDS. Studies have indicated that this herbicide also inhibits SCN. Its mode of action is not fully understood, but data indicates that it may “disrupt the chemoreception and the ability of both nematode species to infect a host root system” [156]. Chemoreception is the process by which organisms respond to chemical stimuli in their environments that depends primarily on the senses of taste and smell.

Michigan State University conducted a study examining the relationship between fluopryam and SCN. Their data indicated that fluopryam decreased the presence of eggs and juvenile nematodes, but did not have an effect on nematode reproduction success. They concluded that fluopryam is better at combatting SCN than no treatment at all, but is not effective enough to be the only strategy used to treat SCN over multiple years [157].

#### General requirements

Soybeans remove larger amount of nutrients from the soil than many other crops; therefore, a higher soybean yield will remove a higher amount of nutrients. The main fertilizer requirements are nitrogen, phosphorus, potassium, sulfur, and iron. Soybeans absorb nitrogen through biological nitrogen fixation, meaning that the addition of nitrogen to the soil will generally have no effect on yield [158].

#### Nutrients and SCN

There has been a large amount of research focusing on the nutritional needs of a variety of nematodes that allow them to thrive in the environments they live in, but the needs of the SCN nematode are yet to be determined. Limiting these nutrients could help combat SCN, but only if they are not already present in the soil. It is possible that removing nutrients that are non-critical to the health of the soybean may make it difficult for SCN to stay alive [159].

## SCN varieties: crop yields in SCN resistant varieties or non-SCN resistant varieties

**Fig. 23:**
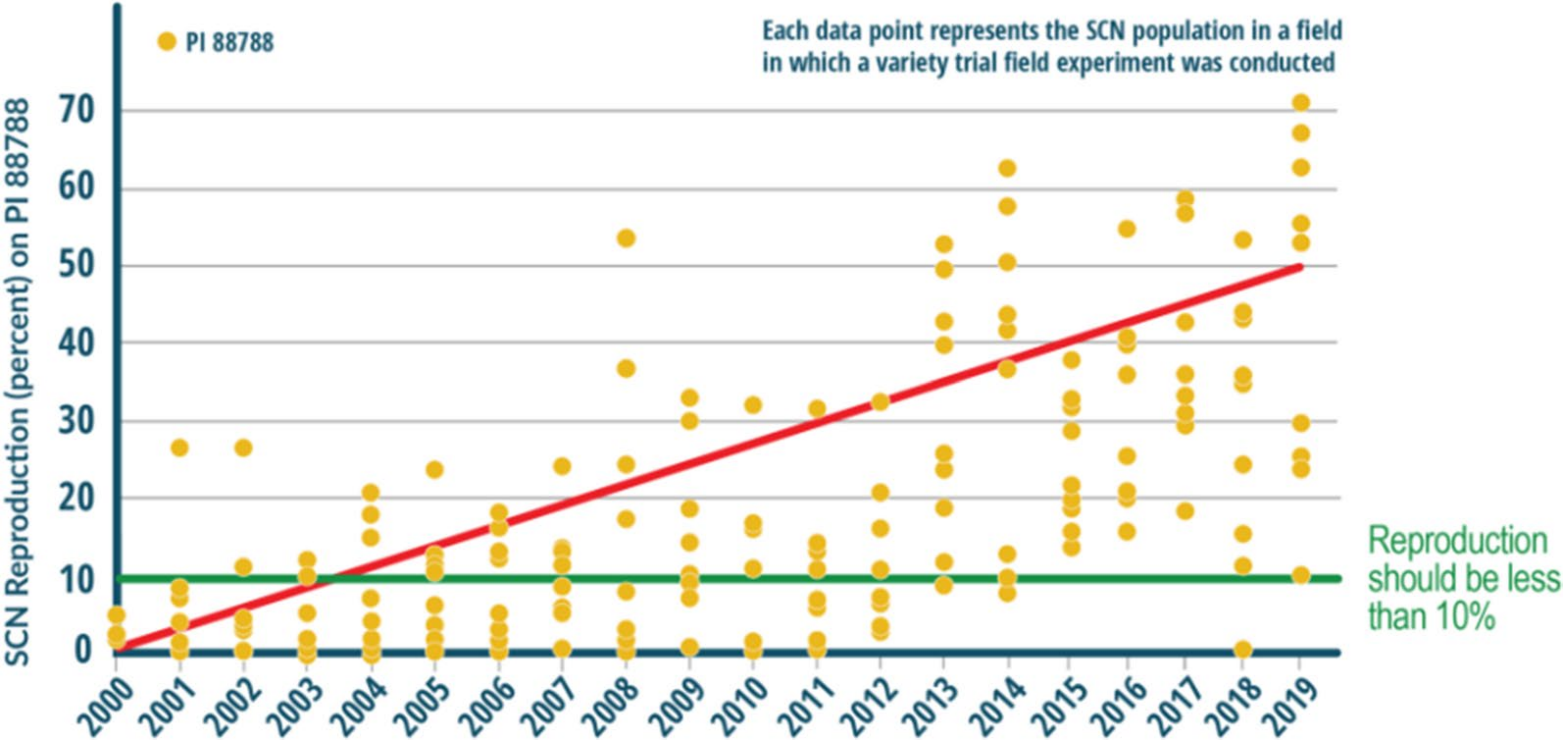
SCN population vs. time (for PI 88788). (source: thescncoalition.com)

One of the key tactics in fighting SCN is using resistant soybean varieties. The three genetic resistant sources that are currently commercially available are PI 88788, PI 548402 (Peking) and PI 437654 (CystX or Hartwig). Each resistant variety uses different tactics to reduce SCN infection. PI 548402 prevents the formation of nurse cells, reducing the nutrient source for a reproductive female. PI 88788 provides poor nutrition for the female, resulting in fewer smaller eggs. SCN resistant soybean varieties stops 90 Farmers are using soybean varieties with the same source of resistance to SCN. PI 88788 was used as a SCN resistant variety for many years. SCN resistant varieties promise a good yield at the beginning of use; however, continuous use of the same strain should not be used for multiple continuous cycles due to potential SCN adaptation. Figure 23 illustrates the adaption of SCN to resistant varieties such as P I88788. Increase in SCN soil population negatively affects yield. It also depicts how the SCN population increase affects yield even when planting soybean resistant variety PI 88788. The reproduction factor is the ratio of number of eggs at the end of the season (Pf) to the number of eggs at the beginning of the season (Pi). An RF value of 40 means the number of eggs in the field increased forty times over the agricultural season.

**Fig. 24:**
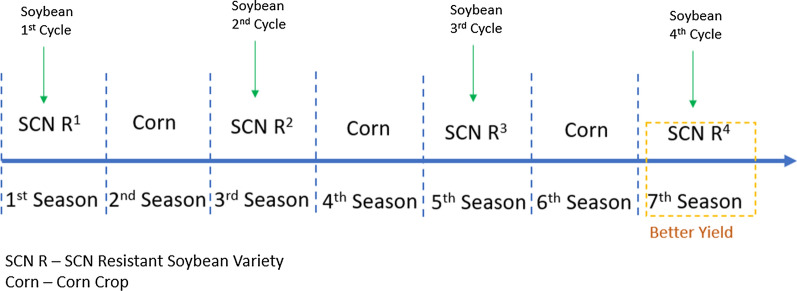
SCN resistant soybean variety rotation scenario

It is highly recommended to rotate the SCN resistant varieties since the SCN population can adapt to individual resistant varieties. Rotating to a different variety may slow the progression of the SCN population. Farmers use a blend of crop rotation along with the rotation of SCN resistant soybean varieties to improve yield (please see Table 15). The rotation of non-host variety crops, such as corn, and SCN resistant varieties are depicted in Fig. 24.

Developing resistant cultivars is the most cost-effective method for managing SCN disease. Different sources of resistance have been discovered but as there are some shifts in SCN populations, which resulted in decrease in resistance from from PI 88788 (from which most of SCN-resistant are derived). SCN are known for their high genetic variations and because of that there are several sources of resistance which have not been fully effective. To mitigate this, a race scheme has been initially used to describe the genetic variations in SCN populations based on four resistant differential lines (1970). The responses to various races are used to classify soybean genotypes. This scheme has been shown to be ineffective in classifying SCN populations. As a result, this scheme has been replaced by a scheme which uses 7 lines instead of 4 [160].

**Table 15:** Varieties of soybean seeds

Company	Variety	Description	Tolerance
Coverta	5J009R2	Branchy group 0, stable performance across all soil and row width.	Phytophthora Root Rot (PRR)
Coverta	5N145R2	Mid-group 1; good height and stability	SCN, Brown stem rot
Coverta	5N157R2	Mid-group 4; Good stability and emergence	Iron deficiency chlorosis, PRR
Coverta	5N207R2	Tall group 2; Good Emergence and Stability; Excellent yield potential	SCN
Growmark/FS	HiSOY 28T42	Group 2.9; Bush bean with strong emergence + early vigor; Light Tawny finish	Sulfonylurea-tolerant, Herbicide flexibility; Moderate white mold pressure.
Growmark/FS	HiSOY 30A42	High yields; Wide adaptability in Northeast soils; Great standability	SCN and PRR
Growmark/FS	HiSOY 36T42	Mid-group 3; moderately productive across Pennsylvania + New Jersey; Light tawny brown; Aggressive early growth	STS/RR2Y Herbicide
Growmark/FS	HiSOY 42T14	Early-group 4; Broad environment + soil type adaption;	Very good SCN, PRR, RR/STS resistance.
Hubner	H34-71XF	Group 3; Tall with great yield potential; Excellent standability; Purple flower color	SCN, Above average Phytophthora tolerance, Sudden Death Syndrome, Southern Stem Canker
Hubner	H32-41XF	Group 3; Tall; Bushy plant; Wide geographical locations	SCN, Above average Phytophthora tolerance, Sudden Death Syndrome, White mold, Brown Stem Rot
Hubner	H35-31XF	Early-Group 3; Excellent performance across wide geographical locations; Excellent standability; Excellent Southern Stem Canker resistance	SCN, PRR Resistance, Brown Stem Rot, Southern Stem Canker, Suddent Death Syndrome
Local Seed Company/Bayer	LS1887X	Late-Group 1; Widely adapted across soil types and environments; Some utility south of zone; Very good standability; Wide + narrow rows	Salt Tolerance, Iron Deficiency, White Mold, Sudden Death Syndrome, SCN (MR3+MR14)
Local Seed Company/Bayer	LS2685X	Mid-Group 2; Medium plant with strong agronomic and disease package; Eastern Environments; Great emergence, standability	Salt Tolerance, Iron Deficiency, White Mold, Frogeye Leaf Spot, Sudden Death Syndrome, SCN (R3+MR14), Phyophthora (Rps1c + 2)
Local Seed Company/Bayer	ZS3296GL	New Early-Group 3; Race horse variety for tighter soils; Perform well under stress	Salt Tolerance, Iron Deficiency, Sudden Death Syndrome, SCN (R3 + MR14), Phyophthora (Rps1c + 0.5), Glyphosate and Glufosinate Tolerance
Local Seed Company/Bayer	LS4495XS	New Mid-Group 4; Highly suited for productive and lodge prone situations;	High Salt Tolerance, Frogeye Leaf Spot, Sudden Death Syndrome, Stem Crank, Phyophthora (Rps1a + 4)
Syngentha	S12-R3	Dominant Performance with soild agronomics; Medium plant height and canopy; Excluder Chloride Sensitivity; Good performance in high pH soil and drought conditions	Brown Stem Rot, Iron Deficiency Chlorosis, Phytophthora Root Rot, Sudden Death Syndrome, Sclerothinia White Mold, Pod & Stem Blight, SCN (R3 + MR14)
Syngentha	S15-3E3	Improved enlist genetics; Medium-tall plant height and canopy; Narrow and wide rows	Iron Deficiency Chlorosis, Phytophthora Root Rot, Sudden Death Syndrome, Southern Stem Canker, Sclerothinia White Mold, Pod & Stem Blight, SCN (R3)
Syngentha	S17-E3	Strong yield and agronomics; Stable performance across environments; Medium plant height + canopy; Includer Chloride Sensitivity; Narrow and wide rows	Iron Deficiency Chlorosis, Phytophthora Root Rot, Sudden Death Syndrome, Sclerothinia White Mold, Pod & Stem Blight, SCN (MR3), Frogeye Leaf Spot

## Discussion and recommendations

### Direct soil sampling

**Fig. 25:**
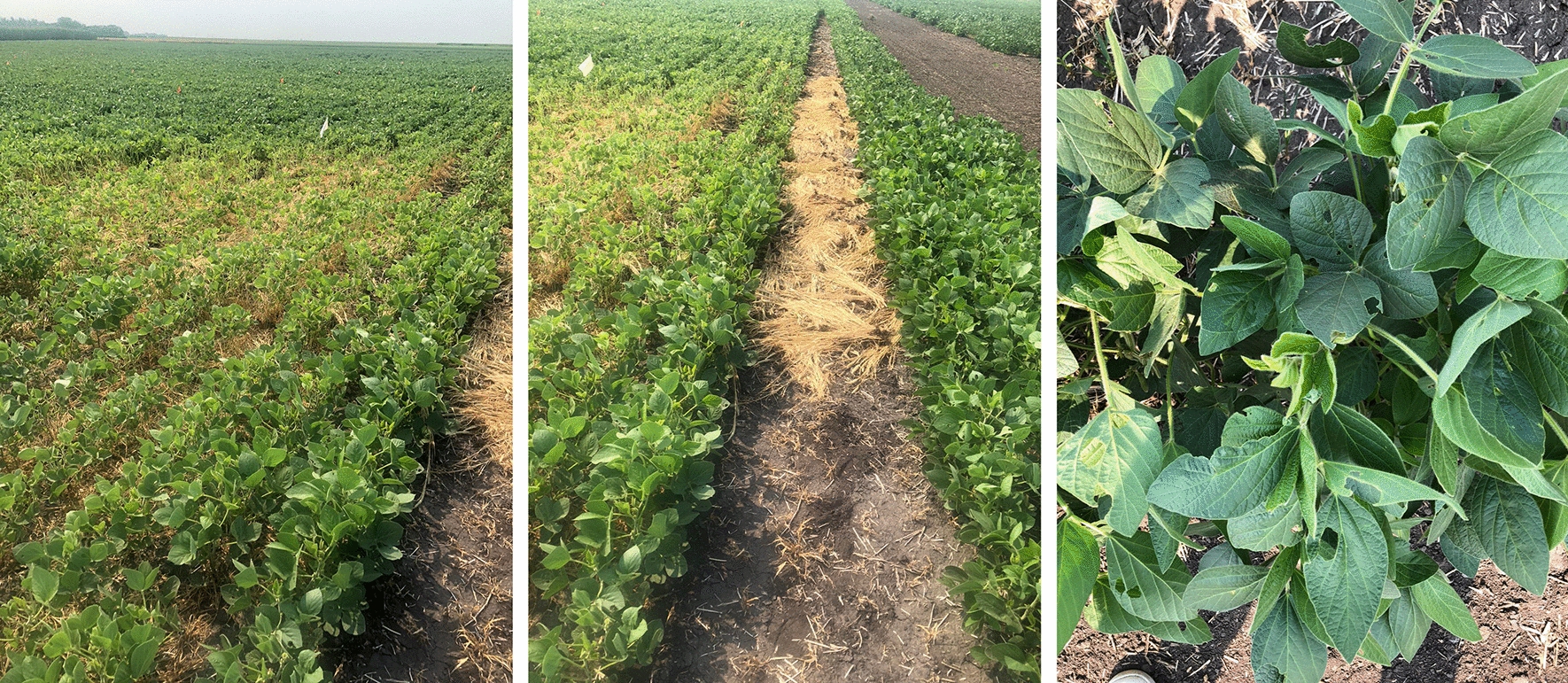
Pictures of Prosper field. Images taken on 2021-07-22. The image to the right shows presence of insect damage to the field

Soil sampling and egg counts remain the most adopted technique used for SCN detection. Collecting soil samples and testing them for egg counts, however, are challenging. Egg counts, for instance, require trained specialists, are time-consuming, and prone to human error; therefore, more than one trained specialist often perform the counts to validate the reported results. Soil sampling and testing precision affect egg count accuracy. As mentioned earlier, the soil testing method used in this study was done by collecting 25 and 42 samples from Casselton and Prosper fields, respectively, where each sample had about 30 cc. of soil. We were informed by experts from the plant diagnostic lab at NDSU that soil samples for SCN egg counts based on the method proposed by [161] which typically requires 250 cc or 100 cc of soil. Taking samples from the field often follow grid methods or taking samples from spots where there are infested soybean. The use of damage threshold $(3~cysts/100~cm^3~soil)$ to deal with SCN is criticised by  many entomologists because  defining a threshold for SCN eggs to make recommendations presents several problems. These problems are listed by the authors of [17]: the SCN population densities at planting and soybean yield vary according to the soil properties and other conditions. The second problem is associated with using cysts as a mechanism to establish the the damage threshold given that the cysts are  not infective units but J2 and assessment of the root infection is still  an unsolved problem. Other information such as P and K levels, soil pH, weather factors should be considered to make reasonable predictions on soybean yield loss but the  relationship between egg numbers, soil properties, weather data, and soybean yield loss remains an open issue. In the absence of an equation that approximates the potential loss damage due to SCN based on information related to SCN egg population density, soil pH, texture, and some other soil factors, weeds and insect pests (see Fig. 25), temperature and rainfall, it is hard to make recommendations to deal with SCN. Therefore, it is very important to develop machine learning techniques or statistical models that can provide such estimates. Even though soil sampling methods and egg counts are  performed at high levels of precision, they are not 100% accurate. If we add to this the issues related to the efficiency of the sampling, then the uncertainty of the results becomes higher. It was reported that the distribution of the eggs in the fields is not uniform so collecting samples that are representative of the field is challenging despite the current recommendations and guidelines. In the absence of soil samples that are not representative of the field, one cannot assess to which extent the SCN has damaged the field. Another issue of direct soil sampling is that the use of tools for extracting soil samples without proper cleaning and disinfecting can help in spreading SCN from fields where SCN exists to fields that are not infested. These challenges have motivated the use of deep learning and imaging techniques to quantify  egg counts and automatically detect SCN without human involvement and in some cases, detecting SCN without going through egg counts and acquiring multispectral/hyperspectral images and study the reflectance spectra to identify infested soybean.

### Deep learning and imaging

Deep learning is a promising approach for soybean cyst nematodes detection and management; the state-of-the-art models indicate adequate performance in detecting soybean diseases or predicting yield loss. Deep learning combined with multispectral imaging, hyperspectral imaging, and data acquired from different sensors such as weather data provides an alternative to direct soil sampling and egg count methods. However, there are still some challenges that need to be addressed to advance this field.

Acquiring hyperspectral imaging using drones is quite new; therefore, there are many questions that are unanswered, such as how to determine accurate resolution the drone is flying at altitude of 10 m since it requires more time to cover the field. Stitching the images together and generating the vegetation indices, using Pix4Dmapper as an example, requires more pre-processing time compared to flying at an altitude of 20:20 m takes approximately 8 min and 10 m takes approximately 20 min to stitch the images and generate the vegetation indexes. There are several other parameters that must be investigated to determine the best combination of parameters in terms of accuracy, cost, and processing time. Assessing the optimal flight parameters for soybean crop management is still an open issue.

Deep learning applications for soybean detection and management is also challenging because of the inherent problems associated with deep learning theory itself, such as overfitting, network hyperparameters tuning, and training time. Deep learning has established the state-of-the-art in the field of hyperspectral imaging; however, training well-generalized models due to a lack of ground-truth data is challenging.

Sometimes, it's not enough to tell farmers that whether they  have SCN fields or not; pointing exactly which parts of the field are infected can help in stopping the SCN spread, especially if detected early. Therefore, deep learning based soybean detection is not enough and methodologies should go multiple steps further by applying instance segmentation on hyperspectral images to not only classify different regions on the field to healthy soybean and infested soybean, but draw bounding boxes around these regions and mapping these regions to GPS coordinates. This is challenging because this methodology requires ground-truth data, which is hard to obtain. To build ground truth data for instance segmentation or semantic segmentation technique, sampling the field using small grid is required. However, sampling the field using small grid is laborious  and requires several days to sample even a small field. Advanced instance and semantic segmentation techniques, such as Mask RCNN, YOLOv4, and Faster RCNN have not yet investigated in soybean disease detection.

It is interesting to investigate deep learning and hyperspectral imaging to assess the root damage caused by SCN. It is quite difficult to perform thi task that why threshold damage methods are based on the cyst and not the root damage. Hyperspectral imaging can provide some tools to assess the root damage but yet there is few to no research papers in this direction even though it can help in detecting SCN more reliably and make recommendations to deal with this damaging pathogen.

Another issue when it comes to deep learning and machine learning research is that there is no straightforward rule for splitting the dataset between training and testing. The most common split percentages are $70{-}30\%$, $90{-}10\%$, and $80{-}20\%$. Some other techniques researchers used $50{-}50\%$. k-Cross validation is another common practice with 5, 10, and 20 folds.

There are several techniques that can be used to alleviate the problem of little ground truth data, such as data augmentation techniques, transfer learning, and unsupervised learning. Data augmentation techniques apply transformations to the images, such as rotation or flipping to augment the number of training samples. Data augmentation presents itself as a good solution to the lack of data. Several data augmentation techniques can be used:

*Adding Salt and Pepper noise* Salt and Pepper noise refers to the addition of white and black dots in the image.

*Rotation (at finer angles)* The network must recognize the object at any orientation. Rotating the image by 90 degrees does not add any background noise if the image is square.

*Flipping* This scenario is more important for networks to remove the bias assumption for certain object features if it is only available on a particular side. Consider the case of an image where the object is a banana tilted to the right. This is certainly not the only orientation that represents this object as such objects can grow or can be captured in different angles. This allows the deep neural network to learn that the  tilt of a banana does not happen only on the  right side. Also, flipping produces different set of images from rotation at multiples of 90 degrees.

*Lighting* Lighting is an important component of the image dataset due to the diversity it creates in images, which is vital for the network to properly learn objects and simulate the practical scenario of the images acquired by the user. The lighting conditions of the images are varied by adding Gaussian noise in the image. The authors of [162] proposed an instance segmentation based on convolutional neural networks and data augmentation applied to spectral images. This approach is useful when there is a lack of ground-truth training data.

Transfer learning techniques use the model trained to perform specific tasks. The random initialization of the weights associated with the connection between the neurons of two successive layers is often used when training a neural network. Random initialization does not help the model converge faster. An alternative is to initialize the set of weights using the set of weights from a pre-trained model, which allows the model to converge faster than with random initialization is used. This technique can be applied to deep learning based on hyperspectral images when detecting soybean cyst nematodes using a set of model weights that have been trained on general features, such as plant reflectance spectra, soil, and water. The model can converge faster and may need fewer training samples. This technique works at it best when the learned features in the first tasks are general. Transfer learning in  image data is successful and can be applied with different CNN models. For instance, the authors of [163] proposed a hyper-spectral image classification using transfer learning. The authors of [164] proposed effective transfer learning for hyper-spectral imaging based on deep learning.

Unsupervised learning-based hyperspectral analysis is another method that can be applied to detect cyst nematodes in soybeans when there is a lack of ground truth data. Unsupervised learning does not typically require labeled training datasets since it clusters the training features to a predetermined number of classes using training dataset sample correlations. A CNN can be used as a feature extraction and feature selection tool before clustering models can be applied to map these extracted features to their corresponding classes. Another approach to tackle the lack of ground training data is the use of unsupervised deep learning models, such as the approach proposed by [165], who investigated the use of fully unsupervised hyperspectral image segmentation.

### Data collection considerations

One of the main challenges associated with UAV-based remote sensing is determining the optimal flight parameters. For instance, at what altitude one should set the vehicles to hover at, whether there is overcast, and suitable wind speeds to prevent any instability or aberrations in image collection; if one or more of these considerations are compromised, the collection and analysis process will be affected. In order to determine the optimal flight parameters, further testing and empirical results are needed. As an example, flying the vehicles using different sets of parameters for each time and studying the impact of these parameters on the accuracy of the detection could be one way to identify  the best set of flight settings. Some other factors come into play such as preprocessing and stitching the image to form the image of whole field, how much overlap exists between images, and how to leverage this information.

### Need for soybean datasets

There are several datasets which can be used in soybean research. For instance, “Soybean (Large) Data Set” [166] is a dataset that contains 19 classes (diaporthe-stem-canker, charcoal-rot, rhizoctonia-root-rot, phytophthora-rot, brown-stem-rot, powdery-mildew, downy-mildew, brown-spot, bacterial-blight, bacterial-pustule, purple-seed-stain, anthracnose, phyllosticta-leaf-spot, alternarialeaf-spot, frog-eye-leaf-spot, diaporthe-pod- &-stem-blight, cyst-nematode, 2–4-d-injury, herbicide-injury) that was created in 1980. It contains more than 30 attributes features such date, plant-stand, precip, temp, hail, crop-hist, area-damaged, etc. a small dataset from Soybean Large Data Set has been derived and known as “Soybean (Small) Data Set” [167]).

The second dataset is a “dataset for weed detection in soybean” [168]. This dataset was used three years ago in Kaggle competition to classify plant images in 4 classes: broadleaf, grass, soil, and soybean. This dataset consists of 15,336 segments: 3249 of soil, 7376 of soybean, 3520 grass, and 1191 of broadleaf weeds.

The third dataset is “Infestation ratings database for soybean aphid on early-maturity wild soybean lines” [169] released by the US department of agriculture to address the issue of soybean aphid (Aphis glycines Matsumura; SA), which is one of the major invasive pest of soybean (Glycine max(L.) Merr.) in northern production regions of North America. “This data set consists of infestation ratings generated for a total of 337 distinct plant introduction lines of wild soybean that were exposed to avirulent SA biotype 1 for 14 d in 25 separate tests”. This dataset was released publicly  to allow for further analyses and evaluation of resistance among the test lines.

The next dataset is related to the soybean price factor data 1962–2018 [170] compilation of soybean prices and factors that effect soybean prices. Temperature columns are daily temperatures of the major U.S. growth areas. Production and area are the annual counts from each country (2018 being the estimates). Prices of commodities are from CME futures and are not  adjusted for inflation. Updates of these CME futures can be found on Quandl. Additional data could be added, such as, interest rates, country currency prices, country import data, and country temperatures.

As it can be seen from the listed examples, there exists no dataset related to soybean cyst nematode detection which can be used for deep learning and imaging. To advance this field, there is a strong need for building large comprehensive datasets that can be used to train deep learning techniques. Having a common public dataset can be used to reproduce the results and assess the proposed deep learning techniques. In addition, scaling up machine learning datasets and building robust detection techniques requires heterogeneity of sources/sensory and data fusion-based approaches to collect datasets. This problem is common to most of ML/DL where hardware and acquisition protocols are different that results in various challenges and incompatibilities between hardware and software. To develop accurate SCN detection, sufficient training data need to be populated. Different geographical factors such as weather, soil type, and seasons in addition to multiple forms of collection i.e., ground-, aerial-, and satellite-based approaches should be considered while collecting these datasets in order to ensure that the models are robust and able to generalize better on unseen data.

### Limitations of computer vision in SCN detection

The following are limitations of detecting SCN using computer vision models:

1. Annotations may need to be completed by nematologists, who are eye trained to distinguish between nematode eggs and non-essential particles such as debris.
2. Imaging defects when using a high precision instrument like a microscope include:
  Different background lighting or orientation of the image sample.
  Inconsistencies in egg colorization during any pre-imaging marking phases.
  Nematode egg obstruction through other microscopic objects.
  Presence of noise, loss of detail, and geometric distortion during digitization degrade image dataset quality.
3. Dataset images must be captured based on the nematode growth stage.
4. Dataset must account for feature distribution differences related to the inherent characteristics of the weather and soil types.
5. Need for protocol of data acquisition to minimize the feature distributions extracted from the images captured by the trained pilots flying the drones.
6. Flying drones sometimes requires optimal weather conditions for better image quality, which may bias the ML/DL models, as few training images that reflect the weather conditions may be present.

Table 16 lists the advantages and disadvantages of direct sampling and computer vision methods for precision agriculture applications.

**Table 16:** Advantages and disadvantages of direct sampling and computer vision methods

Method	Advantages	Disadvantages
Direct sampling	-Widely-used	-Prone to Error
	-Provides Accurate Egg Counts	-Mapping Egg Count, Soil Type, and Nutrients to Presence and Absence of Nematodes is Difficult
Computer vision	-Reduces the Cost and Labor	-Hard to Build Robust Models and Scale the Solution
	-Reduces the Damage Caused to Soybean	-Building Ground Truth for the Dataset by Experts is Challenging
	-Fast	-Absence of Large Training Sets

## Conclusion

Precision agriculture (PA) is a key component of optimized agriculture production to improve the production yield with reduced input losses. PA coupled with the advancements in IoT, ML, and computer vision can make intelligent management decisions to improve crop production. Management strategies for precision crops need spatial, spectral, and temporal knowledge about the crop. Remote sensing techniques are appropriate tools for the derivation of crop parameters. Remote sensing is used to identify, measure, and analyze characteristics of objects of interest without direct contact, and GIS supports storing, analyzing, and retrieving spatially retrieved data. Remote sensing with GIS help farmers and researchers to have a deep understanding of what is happening in the field. Satellites were extensively used for remote sensing before the advancement of autonomous systems such as ground robot and aerial vehicles. Use of satellite for remote sensing gives fast, overall view of the large area while the satellite navigate in it’s orbit. Use of small aircraft or drones gives better sensing capabilities which include better flexibility, flight control, and fast data transfer and hence processing. Sensing using unmanned aerial system (UAS) platforms and aircraft gives a high spatial resolution compared to  the low spatial output from satellite images. UAS platforms allow easy plug and play options while using multiple sensors for investigating a field. In this paper, we have provided a comprehensive review of soybean detection and management techniques with a special focus on machine learning techniques for detecting SCN  and its management. First, we provided an overview on soybean cyst nematode disease and its symptoms as well as its impact on the yield. Then, we provided a classification of SCN detection methods into soil sampling methods and remote sensing methods. We discussed each category and we provided the advantages and the challenges associated with each methodology. We concluded with ongoing research related to deep learning, SCN detection based on hyperspectral imaging, and limitations of machine learning in SCN detection.

## Data Availability

The datasets acquired and analyzed during the current study for this manuscript can be made available from the corresponding author on reasonable request.
